# Loss of proton‐sensing TDAG8 increases tumor progression in mouse models of colon cancer

**DOI:** 10.1002/1878-0261.70283

**Published:** 2026-06-09

**Authors:** Ermanno Malagola, Yasmine Illi, Anna Bircher, Marijn Wilmink, Rachele F. Malagutti, Solenn Ottié, Cordelia Schuler, Pedro A. Ruiz, Cheryl de Vallière, Klaus Seuwen, Gerhard Rogler, Martin Hausmann

**Affiliations:** ^1^ Department of Gastroenterology and Hepatology University Hospital of Zurich, University of Zurich Switzerland

**Keywords:** colorectal cancer, GPR65, inflammatory bowel disease, pH‐sensing G‐protein‐coupled receptors

## Abstract

T‐cell death‐associated gene 8 (TDAG8) is a proton‐sensing G‐protein‐coupled receptor that is activated by neutral to acidic extracellular pH. TDAG8 is primarily expressed in leukocytes and leukocyte‐rich tissues, including the spleen, lymph nodes, and thymus. Genome‐wide association studies have identified TDAG8 as a risk gene for inflammatory bowel disease (IBD). Patients with IBD have an increased risk of developing colorectal cancer (CRC), and TDAG8 has been implicated in regulating anti‐tumor immunity. We used the MC38 and azoxymethane/dextran sodium sulfate (AOM/DSS) mouse models of CRC to evaluate the effect of TDAG8 deficiency on tumor growth. Tumor development was assessed by measuring size and weight, and tumor tissue was analyzed by immunohistochemistry (IHC), real‐time quantitative polymerase chain reaction (qPCR), flow cytometry, and apurinic/apyrimidinic (AP) site quantification. In the MC38 model, *Tdag8*‐deficient mice presented a significantly increased tumor burden together with significantly increased numbers of neutrophils and monocytes in tumor tissue compared with wild‐type (WT) mice. In the AOM/DSS model of CRC, *Tdag8*‐deficient mice exhibited significantly increased tumor size, weight, and number of AP sites compared with WT mice. Additionally, *Tdag8‐*deficient mice showed a significantly increased number of macrophages in tumor tissue and an elevated CD4^+^/CD8^+^ ratio, as confirmed by IHC and flow cytometry. In conclusion, loss of *Tdag8* significantly worsened colon cancer progression in mice and correlated with increased infiltration of tumor‐associated phagocytes. These findings suggest that positive allosteric TDAG8 modulators could represent a promising therapeutic strategy for CRC treatment.

## Graphical abstract

Loss of the pH‐sensing receptor TDAG8 accelerates colorectal cancer progression in mice. Animals lacking TDAG8 expression had increased tumor growth, DNA damage, and recruitment of tumor‐associated immune cells, including macrophages, neutrophils, and monocytes. These findings identify TDAG8 as a protective regulator of tumor‐immune interactions and highlight its potential as a therapeutic target in colorectal cancer.

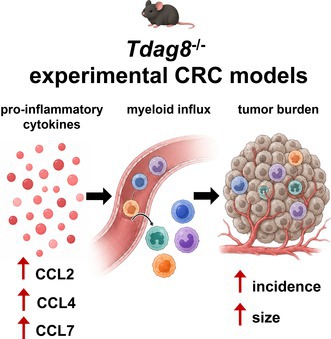

**Abbreviations**
AOM: azoxymethane
AP: apurinic/apyrimidinic
cAMP: cyclic adenosine monophosphate
CD: Crohn's disease
cDNA: complementary DNA
DAB: 3,3′‐diaminobenzidine
DSS: dextran sodium sulfate
ECs: endothelial cells
FSC: forward scatter
*Gapdh*
: glyceraldehyde‐3‐phosphate dehydrogenase
GFP: green fluorescent protein
GI: gastrointestinal
GPRs: G‐protein‐coupled receptors
HBSS: Hank's balanced salt solution
HE: hematoxylin and eosin
IBD: inflammatory bowel disease
IHC: immunohistochemistry
IL: interleukin
KO: knockout
MPO: myeloperoxidase
NK: natural killer
PBS: phosphate‐buffered solution
qPCR: real‐time quantitative polymerase chain reaction
*s.c*.: subcutaneous
SD: standard deviation
SEM: standard error of the mean
SSC: side scatter
TDAG8: T‐cell death‐associated gene 8
UC: ulcerative colitis
WB: Western blot
WT: wild‐type

## 1 Introduction

TDAG8 (GPR65) is a pH‐sensing G‐protein‐coupled receptor that is activated when the extracellular pH drops below physiological levels, reaching maximal activation around pH 6.8, while remaining inactive or almost silent above pH 7.6 [1]. TDAG8 is primarily expressed in leukocytes and in leukocyte‐rich tissues, such as the spleen, thymus, lymph nodes, and small intestine [2, 3, 4, 5, 6].

TDAG8 acts as a regulator of inflammation [7, 8] by activating a Gα_s_‐coupled mechanism [1], which increases downstream cyclic adenosine monophosphate (cAMP) and protein kinase A [3, 9]. In addition, the Rho signaling pathway is activated via G_12/13_ [3]. Rho signaling plays a critical role in controlling leukocyte migration and phagocytosis in innate immune cells.

Inflammatory bowel disease (IBD) encompasses chronic progressive inflammatory diseases of the gastrointestinal (GI) tract—most notably ulcerative colitis (UC) and Crohn's disease (CD)—affecting approximately 1.3 million individuals in Europe (0.2%) and 6.8 million worldwide [10, 11]. IBD is characterized by chronic intestinal inflammation with severe and persistent mucosal damage. Its development is associated with genetic susceptibility [12], alterations in the intestinal microbiota [13], environmental factors [14], and immune dysregulation [15].

A hallmark of intestinal inflammation is local acidification. The progression of IBD is associated with reduced mucosal pH [16, 17]. Stool from patients with severe UC is typically acidic and displays elevated lactate levels [18]. Chronic inflammation promotes tissue acidification through hypoxia and reduced perfusion. The role of TDAG8 in inflammation remains controversial and likely depends on the disease, cell type, and signaling environment. TDAG8 has been reported as both a negative [6, 8, 12, 19, 20, 21, 22, 23, 24] and positive [25, 26] regulator of inflammation.

Genome‐wide association studies have identified TDAG8 as a risk gene in IBD [12]. The single‐nucleotide polymorphism (SNP) rs8005161 is located in a noncoding region [12] and rs3742704 encodes an isoleucine to leucine substitution at position 231 (I231L) of the GPR65 protein [27]. Similar genetic associations have been described in other chronic inflammatory disorders, including chronic obstructive pulmonary disease, asthma, multiple sclerosis, and ankylosing spondylitis [19, 20, 21, 22]. Mechanistically, rs3742704 results in reduced cAMP signaling in response to low pH compared with the reference allele and leads to lysosomal dysfunction, impaired bacterial restriction, and altered lipid droplet formation [27]. Consequently, rs3742704 knock‐in mice are highly susceptible to both bacterial infection‐induced and T‐cell‐driven colitis [28]. In dendritic cells, rs3742704 elevates IL‐12 and IL‐23 release at acidic pH and enhances antigen presentation [28].

In murine models of colitis, *Tdag8*‐deficiency promotes expression of pro‐inflammatory mediators and macrophage infiltration in dextran sodium sulfate (DSS)*‐*induced acute [8] and chronic colitis [6]. In the transfer colitis model, mice receiving naïve *Tdag8*‐deficient T cells exhibit significantly increased spleen weight and enhanced expression of pro‐inflammatory monocyte/macrophage markers [8]. Conversely, pharmacological activation of TDAG8 with an agonist attenuates immune‐mediated inflammation by down‐regulating pro‐inflammatory cytokine production by T cells and macrophages [23].

Patients with UC have approximately a twofold higher risk of developing colorectal cancer (CRC) compared with the general population [29], as well as an elevated risk of hepatobiliary and hematologic malignancies. CD also increases the risk of several cancers, particularly in the upper GI tract, lung, skin, urinary bladder, and blood [11]. These malignancies, together with infections and pulmonary or genitourinary diseases, contribute significantly to IBD‐related mortality [11].

In inflammation‐induced tumors, local acidification is further enhanced by the glycolytic metabolism [30]. Proton (H^+^) accumulation within the tumor microenvironment (TME) results from increased anaerobic and aerobic glycolysis (Warburg effect), leading to lactic acid release and extracellular acidification. In the TME, a local pH below 7.0 is not uncommon and is associated with malignant progression, tumor proliferation, metastasis, metabolic rewiring, and impaired immune surveillance [31, 32, 33, 34].

The role of TDAG8 in cancer is complex and context‐dependent. It has been described as both a negative [35, 36] and positive [6, 37] modulator of tumor development. TDAG8 expression is markedly reduced in human blood cancers, and its restoration suppresses tumor growth *in vitro* and in murine xenograft models [35]. The coding region SNP rs3742704 is associated with improved survival across multiple cancer types [36]. In contrast, TDAG8 inhibition *in vitro* has been shown to enhance T‐cell anti‐tumor activity [36].

In colitis‐associated CRC, TDAG8 appears to exert a protective role. In the azoxymethane (AOM)/DSS model, which recapitulates early tumorigenesis, Tdag8‐deficient mice develop a higher number and larger size of colonic tumors compared with wild‐type (WT) controls [6]. Conversely, overexpression of TDAG8 in mouse Lewis lung carcinoma cells enhances tumor development [37], suggesting that its function may depend on the cellular or microenvironmental context.

The present study aimed to clarify the physiological role of TDAG8 in colon tumorigenesis. To this end, we employed both the AOM/DSS model of inflammation‐driven colorectal cancer and the MC38 syngeneic subcutaneous (*s.c*.) tumor model in *Tdag8*‐deficient and WT mice. Our results demonstrate that loss of functional TDAG8 significantly enhances colon cancer growth, accompanied by increased CD4^+^/CD8^+^ ratios and elevated numbers of neutrophils and monocytes within the tumor tissue.

## 2 Materials and methods

### 2.1 Animals

All animal experiments were performed according to the ARRIVE criteria. *Tdag8* ^*−/−*^ mice (B6‐Gpr65< tm1Dgen>) were generated by Deltagen (San Mateo, Ca, USA) [38]. The animal experiment protocol was approved by the Veterinary Authority of the canton of Zurich (registration number ZH211/2020 and ZH113/2021). Mice with an identical genetic background (littermates) were used in all experiments.

For tumor induction with AOM/DSS, six female WT mice were compared with five female *Tdag8* ^*−/−*^ mice. For tumor induction with MC38 (RRID:CVCL_B288, a murine colon adenocarcinoma cell line), 15 WT mice were compared with 13 *Tdag8* ^*−/−*^ mice. In both experiments, female mice aged 10–13 weeks and weighing 21.9 ± 1.7 and 22.4 ± 1.0 g, respectively, were used. To minimize aggression under stressful experimental conditions, only female mice were used.

The animals were co‐housed whenever possible, and bedding was exchanged among the cages to minimize microbiota variation. All the animals were housed in a specific pathogen‐free facility as previously described [39]. The animals were kept in type II long clear‐transparent individually ventilated cages (IVCs, 365 mm × 207 mm × 140 mm, Allentown, New Jersey, USA) with autoclaved dust‐free bedding and tissue papers for nesting. Mice were fed a pelleted and extruded mouse diet (R/M–H Extrudat, ssniff Spezialdiäten, Soest, Germany) *ad libitum*. The light/dark cycle was provided through natural daylight (sunrise: 07:00 h, sunset: 18:00 h). Animals were weighed at 10:00 h every morning. The conditions were maintained at 21 ± 1 °C, relative humidity of 55 ± 5% and 75 complete changes of filtered air per hour (filter: Megalam MD H14, Camfil, Zug, Switzerland).

### 2.2 Tumor models

The AOM/DSS model of colitis‐induced cancer was performed according to a modified protocol by Neufert *et al*. [40]. Briefly, colitis was induced with DSS (36–50 kDa, MP Biomedicals, Santa Ana, CA, USA) as previously described [41]. For chronic colitis WT and *Tdag8* ^−/−^ mice were administered with four cycles of 3% DSS in drinking water *ad libitum* for 7 days, followed by 10 days of normal drinking water. To induce tumor growth, AOM (Sigma‐Aldrich, 10 mg/kg in saline) was injected intraperitoneally (*i.p*.) on Day 1 and Day 9 of each DSS cycle. Body weight and clinical phenotype were monitored daily. Colonoscopy was performed under anesthesia before the third DSS cycle and at the end of the experiment to assess tumor development and inflammation. After the last DSS cycle, all animals were allowed to recover for 3 weeks and before sacrifice and tissue sampling.

MC38 cells (RRID:CVCL_B288) were originally obtained from Dr. J. Schlom (NIH) in 1997, expanded to low passage number and frozen as working stocks [42]. For injection models, MC38 and MC38_luciferase (RRID:CVCL_C8VZ, both cell lines donated by Lubor Borsig, Institute of Physiology, University of Zurich, Zurich, Switzerland [42]) were used. All cell lines were tested using the EZ‐PCR Mycoplasma Kit (20‐700‐20, Lucerna Chem AG, Switzerland) and found to be free of mycoplasma; no further cell authentication assays were performed. Cells were passaged two to three times after thawing prior to implantation into mice.

For *s.c*. tumor induction, 300 000 cells per tumor were suspended in culture medium, mixed 1:2 with Matrigel, and injected into the flank of mice. Tumor growth was measured every 2 days using a digital caliper. Tumor volume was calculated using the ellipsoid formula: 4/3 × 3.14 × length/2 × (width/2)^2^, where the shorter dimension was used as width and depth. Experiments were terminated according to the veterinary office: All mice in an experimental group were euthanized before the tumors of one or more animals in the experimental group reached a volume of 1 cm^3^ or a length of 2 cm. Mice with MC38 and MC38‐luciferase were euthanized 14 days postinjection. A total of 10 WT mice were compared with 10 *Tdag8* ^−/−^ mice using MC38_luciferase cells. A total of 5 WT mice were compared with 3 *Tdag8* ^−/−^ mice using MC38 cells.

### 2.3 Luciferase assay

At the end of the experiment, mice bearing MC38_luciferase cells were anesthetized, and luciferase assay was performed to monitor tumor development prior to sacrifice. Five tumors from WT mice were compared with five tumors from *Tdag8* ^−/−^ mice. Mice were incubated with Xenolight D‐Luciferin (Perkin Elmer, Waltham, MA, USA, 122799) for 10 min at 37 °C. Luciferase activity was analyzed using IVIS Spectrum Imaging (Zurich Integrative Rodent Physiology, University of Zurich).

### 2.4 Assessment of colonoscopy and histological score in mice

Prior to endoscopic assessment, the animals were anesthetized *i.p*. with a mixture of 90–120 mg/Kg body weight ketamine (Narketan 10%, Vétoquinol AG, Bern Switzerland) and 8 mg/Kg body weight xylazine (Rompun 2%, Bayer, Switzerland), and examined with the Tele Pack Pal 20 043 020 (Karl Storz Endoskope, Tuttlingen, Germany) as previously described [43].

For histological scoring, colon and small intestine were prepared as Swiss rolls, fixed in paraformaldehyde solution (4% in PBS, Santa Cruz Biotechnology, USA), embedded, stained with hematoxylin and eosin (HE), and scored as described [44]. Mice were scored individually. Histology was performed by an independent investigator blinded to treatment. Histology scoring criteria were applied as previously described [39]:

Epithelium (E) 0: normal morphology; 1: loss of goblet cells; 2: loss of goblet cells in large areas; 3: loss of crypts; 4: loss of crypts in large areas

Infiltration (I) 0: no infiltrate; 1: infiltrate around the crypt base; 2: infiltrate extending to the *L. muscularis mucosae*; 3: extensive infiltrate extending to the *L. muscularis mucosae* and mucosal thickening with abundant edema; 4: infiltration of the *L. submucosa*.

The total histological score was calculated as the sum of epithelial and infiltration scores, ranging from 0 to 8 (total score = E + I).

### 2.5 Immunohistochemistry (IHC) and immunofluorescence

Specimens were fixed in 4% PBS‐buffered formalin, embedded in paraffin and sectioned (3 μm) as previously described [39]. HE staining was performed using standard procedures. Tissues for IHC were deparaffinized, and antigen retrieval was performed in citrate buffer (pH 6.0, Dako) at 98 °C for 30 min. Endogenous peroxidase activity was inhibited with 0.9% hydrogen peroxide for 15 min at RT. Nonspecific binding was blocked with serum for 1 h at RT. Primary antibodies were diluted in blocking solution and slides were incubated overnight at 4 °C. For IHC samples, a secondary HRP‐conjugated antibody (Vector Laboratories, Newark, CA, USA, ImmPRESS) was applied for 1 h at RT, and staining was visualized using the 3,3′‐diaminobenzidine (DAB) ImmPACT Peroxidase Substrate Brown Kit (Vector Laboratories). IHC specimens were counterstained with hematoxylin, dehydrated, and mounted. F4/80 expressed by macrophages was stained with monoclonal rat anti‐mouse antibody (T‐2006; BMA Biomedicals AG, Augst, Switzerland, 1:50). MMP9 was stained with a monoclonal mouse anti‐mouse MMP9 (NBP2‐13173; Novus Biologicals, Centennial, CO, USA, 1:200) antibody. ATAD2 was stained with a monoclonal rabbit anti‐mouse ATAD2 (50563S; Cell Signaling, Danvers, MA, USA, 1:200) antibody. STMN1 was stained with a polyclonal rabbit anti‐mouse STMN1 (11157‐1‐AP; Thermo Fisher Scientific, Waltham, MA, USA, 1:200) antibody. IHC for CXCL12/SDF‐1 was performed on a Ventana stainer (Roche, Basel, Switzerland) with a monoclonal mouse anti‐mouse (MAB350; R&D Systems, Minneapolis, MN, USA, 1:100), CK20 with a monoclonal rabbit anti‐mouse (ab64909; Abcam, Cambridge, UK, 1:100). Specimens were stained with hematoxylin using standard histological techniques. Staining was examined using the Imager Z2 microscope and the software ZEN (Carl Zeiss). Images were acquired at 20x magnification from at least three representative areas per section using transmission light microscopy. Relative quantification of antigen expression was performed with the Fiji software (1.52a, NIH). Total tissue area was determined by converting the image into an 8‐bit format and adjusting the B&W threshold. The area covered with DAB was determined setting thresholds for brown coloration and antigen expression was calculated as the ratio of DAB positive area to total tissue area.

### 2.6 RNA isolation, complementary DNA (cDNA) synthesis, and qPCR

Total RNA was isolated from colon and ileum using the Maxwell RSC simplyRNA tissue kit (Promega, Madison, WI, USA, AS1340) as previously described [43]. Lysis buffer was added to snap‐frozen resections, and samples were homogenized in M tubes (Miltenyi Biotec, Bergisch Gladbach, Germany) using a gentleMACS tissue homogenizer (Miltenyi Biotec). RNA concentration was determined by absorbance at 260 nm and 280 nm with a NanoDrop (Thermo Fisher Scientific, Waltham, MA, USA). cDNA was synthesized with the High‐Capacity cDNA Reverse Transcription Kit (Applied Biosystems, Foster City, CA, USA) following the manufacturer's instructions. qPCR was performed using the TaqMan Fast Universal Master Mix (Applied Biosystems) on a QuantStudio™ 6 Flex Real‐Time PCR System and data were analyzed with the SDS software (Applied Biosystems). Each sample was measured in triplicates, with glyceraldehyde‐3‐phosphate dehydrogenase (*Gapdh*) serving as the endogenous control. Results were analyzed using the ∆∆CT method. The following mouse gene expression assays (Thermo Fischer Scientific) were used: *Ccl2* mm 00441242_m1, *Ccl3* Mm00441259_g1, *Ccl4*, mm00443111_m1, *Ccl7* Mm00443113_m1, *Il‐6* mm00446190_m1, *Tnf* mm99999068_m1, and *Gapdh* 4352339E.

### 2.7 Quantification of AP sites

DNA damage was determined using a colorimetric assay kit (ab211154; Abcam, Cambridge, UK) according to the manufacturer's instructions. Briefly, snap‐frozen whole colon resections from the AOM/DSS CRC model were disrupted in 400 μL lysis buffer in M tubes (Miltenyi Biotec, Bergisch Gladbach, Germany) using a gentleMACS tissue homogenizer (Miltenyi Biotec). Genomic DNA concentration was determined by absorbance at 260 nm and 280 nm using a NanoDrop spectrophotometer (Thermo Fisher Scientific, Waltham, MA, USA). Purified genomic DNA (100 μg/mL) was diluted in TE buffer, and 5 μL was combined with 5 μL aldehyde reactive probe solution. DNA was precipitated with glycogen, sodium acetate, and absolute EtOH at −20 °C for 30 min. After centrifugation, the DNA pellet was washed three times with 70% ethanol, air dried, and resuspended in TE buffer (1 μg/mL). DNA binding solution, streptavidin‐enzyme conjugate, and substrate solution were added to a 96‐well plate and incubated at room temperature for ≤ 20 min on an orbital shaker. Stop solution was added, and absorbance was measured at OD 450 nm on a microplate reader. The OD values, corrected for the absorbance value of the blank, were applied to the standard curve to calculate the number of AP sites per 10^5^ bp.

### 2.8 Western blot

Equal amounts of protein were separated by SDS/PAGE and transferred for immunoblotting. Membranes were probed with monoclonal rabbit anti‐mouse MPO (ab208670; Abcam, Cambridge, UK, 1:1000), polyclonal rabbit anti‐mouse β‐actin (#4970, 13E5; Cell Signaling, Danvers, MA, USA, 1:2000) and the horseradish peroxidase‐conjugated goat anti‐rabbit secondary antibody (#sc‐2004; Santa Cruz, CA, USA, 1:2000). Luminescence was quantified densitometrically with Image J.

### 2.9 Tumor cell isolation

Tumor was minced into small pieces and digested in 6 mL HBSS (Sigma, Hanks' Balanced Salts, H2387‐10X1L), supplemented with 0.5 mg/mL collagenase type IV (Gibco, 17 104–019) and 0.05 mg/mL DNaseI (Roche, 10 104 159 001) for 15 min at 37 °C with shaking. After incubation, samples were sheared using a syringe fitted with an 18‐G needle and filtered through a 70‐μm cell strainer. Digestion was stopped by adding 3 mL low Ig (PAN Biotech, P30‐2802) fetal bovine serum (FBS) and 10 mL PBS. The supernatant was removed after centrifugation at 1500 rpm for 5 min at 4 °C.

### 2.10 Flow cytometry

For single‐cell flow cytometry analysis, single cells from tumors were isolated. Surface antigens were stained with a mix of antibodies, including a viability marker (Table S1), and incubated at 4 °C for 20 min as previously described [39]. After washing with PBS and centrifugation, all the samples were fixed with BD Cytofix/Cytoperm (554722) and BD Perm/Wash (554723) following the manufacturer's instructions. Pellets were resuspended in PBS and intracellular antigens were stained with a mix of antibodies (Table S1). Data were acquired on a FACS LSR II Fortessa 4 L (BD) and analyzed with the FlowJo software (version 10.2).

### 2.11 Compensation controls

Compensation controls were prepared using two beads kits as previously described [39]. The ArC Amine reactive compensation bead kit (A10346; Thermo Fisher Scientific) was used for the cell viability marker. For the remaining antibodies, BD CompBeads kit anti‐Rat and anti‐Hamster (552 845; Becton Dickinson Pharmingen Biosciences), and the BD CompBeads anti‐Mouse (552 843; Becton Dickinson Pharmingen Biosciences) were used. All the compensation controls were prepared following the manufacturer's instructions.

### 2.12 Single‐cell RNA sequencing (scRNAseq) analysis

Single cells were isolated from tumors of a WT mouse upon the AOM/DSS model of colitis‐induced cancer. Debris was removed with Debris Removal Solution (130‐109‐398; Miltenyi Biotec, Bergisch Gladbach, Germany). scRNAseq was performed as previously described [45]. Briefly, for droplet sequencing, cells were pelleted at 3000 × ***g*** for 10 min at 4 °C, resuspended in 1X PBS with 1% BSA (Thermo Fisher Scientific) and 0.5 U/μL RNasin (Promega, Madison, WI, USA), and filtered through a 40‐μm Flowmi Cell Strainer (Sigma‐Aldrich). From each mouse, 10 000 cells (maximum recovery) were loaded onto an individual lane of a 10X Genomics Chromium Single Cell microfluidics chip using v3 chemistry (10X Genomics; Pleasanton, CA, USA). Libraries were pooled and sequenced on an Illumina NovaSeq 6000 platform (Illumina, San Diego, CA, USA) at the Functional Genomics Center of the University of Zurich/ETH Zurich.

Single‐cell transcriptomic data were analyzed using Seurat v5.4 following standard computational workflows. Cells were filtered for quality control, selecting only high‐quality cells (nFeature_RNA > 500 & nFeature_RNA < 5000 & nCount_RNA > 2000 & nCount_RNA < 70 000 & percent.mt < 20). Following clustering and dimensional reduction analyses, differential gene expression analysis was performed to identify cluster markers was performed using the FindAllMarkers function (‘wilcox’ test). Consequently, cells were grouped into five major clusters based on selected marker genes expression. Publicly available datasets of human CRC patients (GSE132465) were analyzed based on authors’ published annotations (‘Cell_subtype’) [46].

### 2.13 Statistical analysis

Statistical analysis was performed as indicated in the figure legends using GraphPad Prism (v 9.4.1), as previously described [39]. Results are presented as mean ± standard deviation (SD) or ± standard error of the mean (SEM), as indicated in the figure legends. Differences were considered significant at *P* < 0.05 (*), highly significant at *P* value of < 0.01 (**), and very highly significant at *P* value < 0.001 (***).

## 3 Results

### 3.1 *Tdag8* is predominantly expressed in T cells

**Fig. 1:**
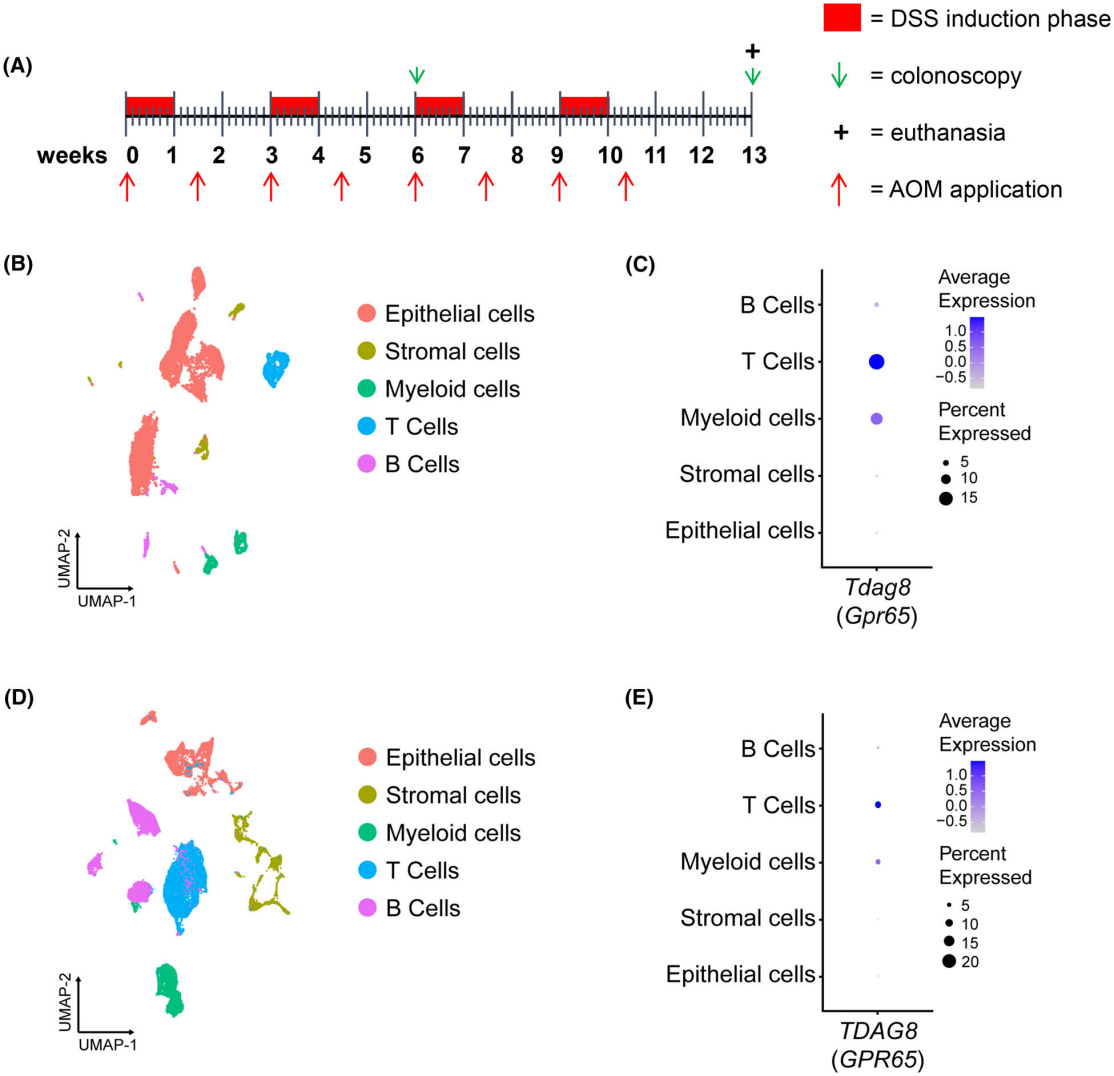
TDAG8 is predominantly expressed in T cells. Murine and human CRC analysis. (A) Experimental timeline for tumor induction with azoxymethane/dextran sodium sulfate (AOM/DSS). (B) UMAP plot of murine colonic tumor tissue showing major cell populations. (C) Dot‐plot showing expression levels of murine *Tdag8* across major cell types. Dot color indicates average expression, and dot size reflects the percentage of cells expressing the gene. (D) UMAP plot of human colonic tumor tissue (scRNAseq data from [46]). (E) Dot‐plot showing expression levels of human *TDAG8* across major cell types.

To investigate the role of TDAG8 in CRC, we first examined its expression pattern in the AOM/DSS model, a spontaneous model of tumorigenesis that mirrors early stages of tumor development, with tumors appearing within 10–12 weeks after treatment onset (Fig. 1A).

Following colonoscopy and tissue harvest, macroscopically visible tumors were dissected and processed for scRNAseq. We obtained approximately 10′000 high‐quality cells that clustered into multiple populations representing colonic epithelial cells, stromal cells, and immune infiltrates (Fig. S1A,B). Known markers of early colorectal tumorigenesis, including *Notum* [47] and *Tacstd2* [48], were detected, confirming the presence of early neoplastic lesions within the colonic epithelium of AOM/DSS‐treated mice (Fig. S1C).

Cells were subsequently grouped into major categories—epithelial, stromal, myeloid, T, and B cells (Fig. 1B)—and *Tdag8* expression was analyzed across these populations. *Tdag8* was predominantly expressed in T cells, followed by myeloid and B cell clusters, with minimal expression in epithelial or stromal compartments (Fig. 1C).

To determine whether this pattern was conserved in humans, TDAG8 expression was examined in a publicly available dataset of resected human CRC and adjacent nontransformed tissue [46]. Consistent with the murine data, TDAG8 expression was highest in T cells, followed by myeloid and B cell populations (Fig. 1D–E).

Overall, these data indicate that within colon tumors, *Tdag8* expression is confined to the immune infiltrate, particularly T cells, with minimal to no expression detected in epithelial or stromal cells.

### 3.2 *Tdag8* deficiency increases intestinal inflammation in the AOM/DSS model

Inflammation is a critical modulator of colorectal tumorigenesis [49, 50]. Previous studies have shown that TDAG8 protects against excessive inflammation in response to diverse insults. Based on this, we hypothesized that TDAG8 deficiency would exacerbate inflammation and accelerate tumor formation in the AOM/DSS model of colitis‐associated cancer.

**Fig. 2:**
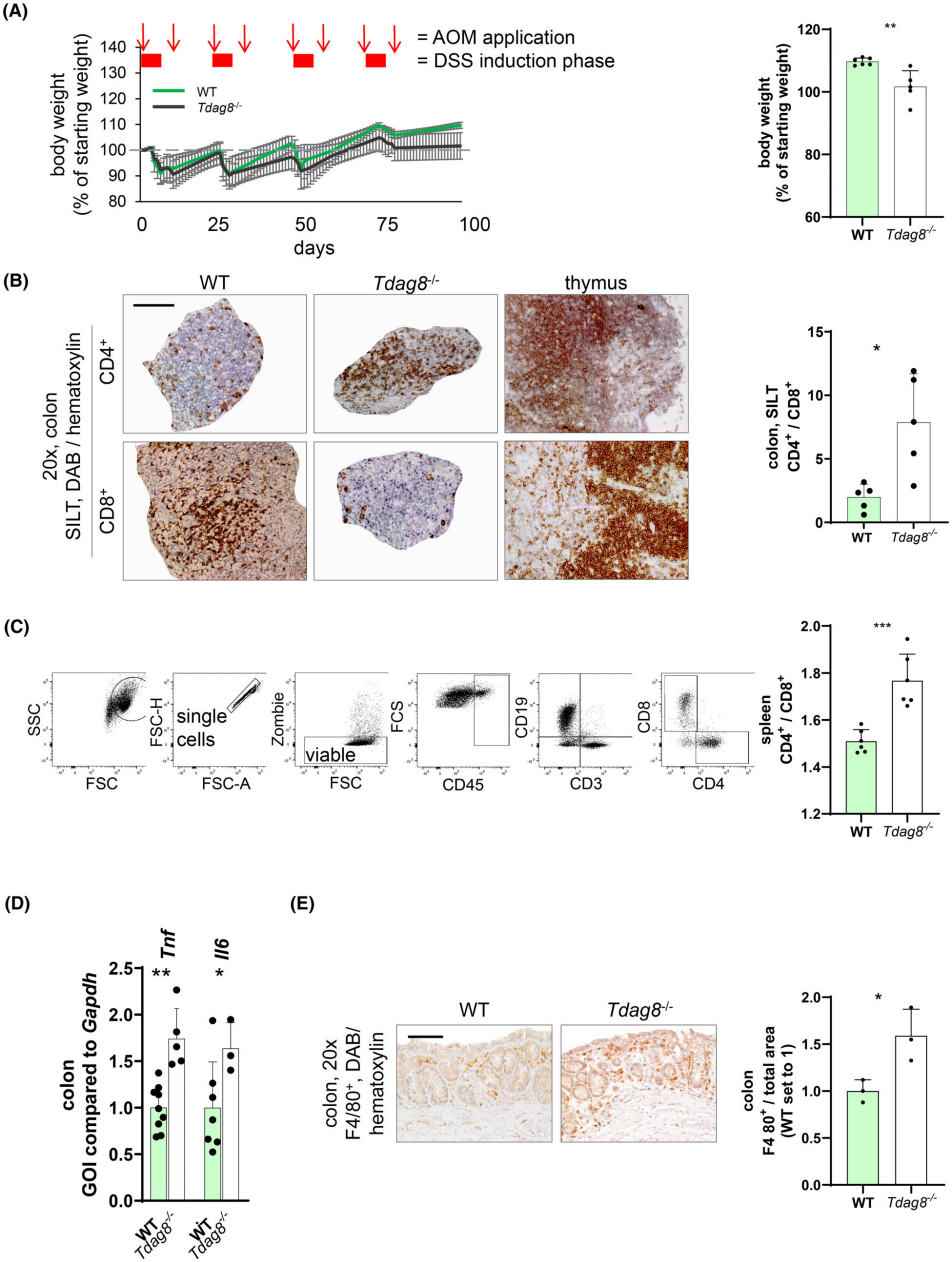
Increased inflammation in *Tdag8* ^−/−^ compared with WT mice upon AOM/DSS colitis. (A) Body weight and quantification (*P* = **, *n* = 6 mice for WT and 5 mice for *Tdag8* ^−/−^). (B) IHC in SILTs and quantification, CD4^+^ and CD8^+^ T cells. Scale bar for 20 × 100 μm (DAB brown, hematoxylin blue, *P* = *, *n* = 5 mice for WT and 5 mice for *Tdag8* ^−/−^). (C) Flow cytometry and quantification, CD4^+^ and CD8^+^ T cells in spleen (*P* = ***, *n* = 6 spleens for WT and 6 spleens for *Tdag8* ^−/−^). (D) qPCR quantification, *Tnf* (*P* = ***, *n* = 9 mice for WT and 5 mice for *Tdag8* ^−/−^) and *Il6* (*P* = 0.0739, *n* = 7 mice for WT and 3 mice for *Tdag8* ^−/−^). (E) IHC and quantification, F4/80, macrophages. Scale bar for 20x 100 μm (DAB brown, hematoxylin blue, *P* = *, *n* = 3 mice for WT and 3 mice for *Tdag8* ^−/−^). Normal distribution (Shapiro–Wilk test), unpaired *t*‐test. Error bars indicate SD. WT, wild‐type; DAB, 3,3′‐diaminobenzidine; AOM, azoxymethane; DSS, dextran sodium sulfate; SILT, solitary intestinal lymphoid structure.

To test this, we compared tumor development in six WT mice and five *Tdag8* ^*−/−*^ mice in the AOM/DSS model. *Tdag8* ^*−/−*^ mice exhibited a significant body weight loss (Fig. 2A), consistent with impaired regulation of inflammation. Despite these observations, spleen weight and histological scores of the colon and small intestine were not significantly altered (Fig. S2A–C).

Because the CD4^+^/CD8^+^ T‐cell ratio reflects the balance between pro‐inflammatory helper and cytotoxic T‐cell responses, we next examined whether TDAG8 loss altered this balance. IHC revealed an increased CD4^+^/CD8^+^ ratio in solitary intestinal lymphoid tissue (SILT) of *Tdag8* ^*−/−*^ mice compared with controls (Fig. 2B) and this was confirmed by flow cytometry of splenocytes (Fig. 2C, Table S1, panel 1). This elevation in the CD4^+^/CD8^+^ ratio indicates a shift toward a pro‐inflammatory state in *Tdag8* ^*−/−*^ mice.

To further determine whether loss of Tdag8 predisposes toward the establishment of a pro‐inflammatory state within colonic tumors, we investigated the expression of multiple pro‐inflammatory cytokines. *Il6* [51] and *Tnf* [52] are key mediators of macrophage activation; moreover, Increased IL6 [53, 54] and TNF [55] induce chemokine signaling cascades which allow the recruitment of macrophages. Notably, both *Il6* and *Tnf* expression was found to be predominantly associated to the myeloid cell cluster, in both murine and human tumors, along with *Ccl3*, another known pro‐inflammatory cytokine (Fig. S3A).

Expression of the pro‐inflammatory cytokines *Tnf* and *Il6* was significantly increased in colonic tissue from *Tdag8* ^*−/−*^ mice compared with WT controls (Fig. 2D). To determine whether these cytokine changes were accompanied by increased macrophage infiltration, we quantified F4/80^+^ cells, a well‐established marker of tissue‐resident and infiltrating macrophages. IHC demonstrated a significant increase in F4/80+ macrophages within the lamina propria of *Tdag8* ^*−/−*^ colonic tissue (Fig. 2E; Fig. S3B), indicating enhanced macrophage recruitment and an amplified inflammatory response in the absence of TDAG8. Together, these findings support a protective role of TDAG8 in limiting intestinal inflammation during colitis‐associated tumorigenesis.

### 3.3 *Tdag8* deficiency increases tumor burden in the AOM/DSS model

To assess the impact of TDAG8 loss on colon tumorigenesis, we analyzed tumor development in *Tdag8* ^*−/−*^ and WT mice using the AOM/DSS model. Progressive colonoscopy revealed early dysplastic lesions in both genotypes prior to the third DSS cycle (Fig. S4A).

**Fig. 3:**
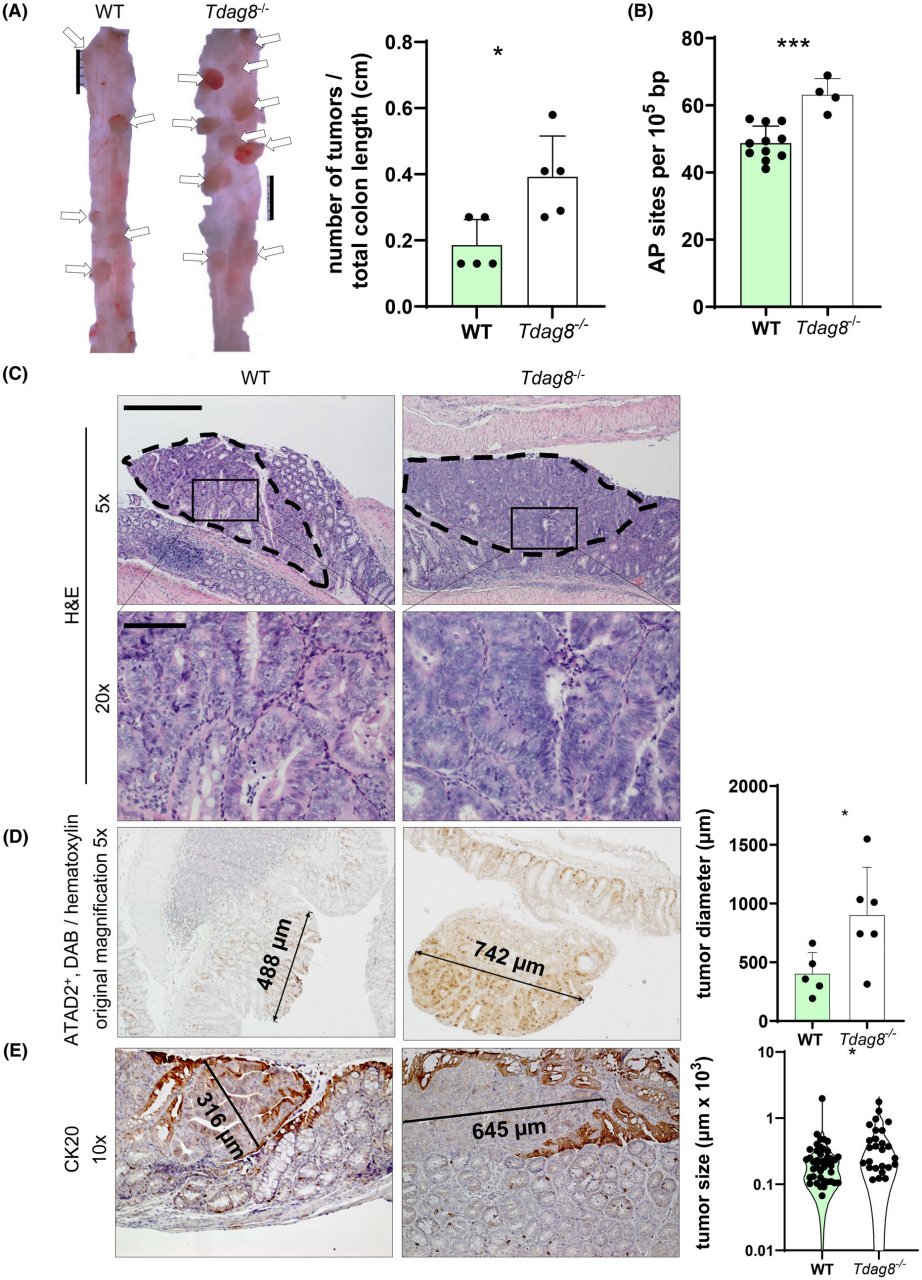
Increased number of tumors in *Tdag8* ^−/−^ compared with WT mice upon AOM/DSS colitis. (A) Macroscopic mucosal image of the colon after longitudinal incision taken with stereo microscope. Merged images. Scale bars 5 mm. Number of tumors per total colon length (in cm), and quantification (*P* = *, *n* = 5 mice for WT and 5 mice for *Tdag8* ^−−/−^, the statistics include 69 tumors). (B) AP sites, colon, and quantification (*P* = ***, colon resections from *n* = 11 mice for WT and 4 mice for *Tdag8* ^−−/−^). (C) H&E. Scale bar for 5 × 500 μm and 20 × 100 μm. Dashed line = tumor, representative images. (D) IHC and quantification, ATAD2, tumor size. Scale bar as indicated (DAB brown, hematoxylin blue, *P* = *, *n* = 5 tumors from 5 mice for WT and 6 tumors from 5 mice for *Tdag8* ^−/−^, the statistics include 11 tumors). (E) IHC and quantification, CK20, tumor size. Scale bar for panorama 1 mm, for 10 × as indicated (*P* = *, *n* = 42 tumors from 5 mice for WT and 26 tumors from 5 mice for *Tdag8* ^−−/−^). Normal distribution (Shapiro–Wilk test), unpaired *t*‐test. Error bars indicate SD. WT, wild‐type; DAB, 3,3′‐diaminobenzidine; AOM, azoxymethane; DSS, dextran sodium sulfate; AP, apurinic/apyrimidinic.

*Tdag8* ^−/−^ mice developed a significantly higher number of tumors compared with WT controls (Fig. 3A), while colon length remained unchanged (Fig. S4B). DNA damage, assessed by AP sites, was also significantly increased in *Tdag8* ^−/−^ colonic tissue (Fig. 3B), indicating decreased genomic stability within lesions.

Histological analysis by HE staining confirmed pronounced epithelial abnormalities in both genotypes, with *Tdag8* ^*−/−*^ mice exhibiting extensive dysplasia and tumor formation (Fig. 3C). The lesions showed distorted crypt architecture and loss of mucosal organization, features characteristic of colitis‐associated neoplasia.

To objectively quantify the extent of epithelial transformation, we devised a system to measure lesion area across tissue sections using markers of epithelial stemness previously identified in murine small intestinal epithelium [56]. ATAD2 (Fig. 3D) and STMN1 (Fig. S5A) reliably labeled dysplastic regions, enabling clear distinction between transformed and adjacent nontransformed tissue. Quantitative analysis of ATAD2^+^ areas revealed significantly larger tumor diameters in *Tdag8* ^*−/−*^ mice, indicating that loss of TDAG8 not only increases tumor incidence but also promotes lesion expansion. In normal tissue, ATAD2 expression was confined to the crypt base and did not differ between genotypes (Fig. S5B). Similarly, STMN1 expression was restricted to proliferative regions and was increased within dysplastic or tumorous areas of both groups (Fig. S5A–C).

We further validated this approach using markers of epithelial differentiation. CK20, an established marker widely used for the histopathological identification of human colon adenocarcinomas [57], clearly delineated tumor margins, defining the boundary between neoplastic and adjacent normal mucosa (Fig. 3E; Fig. S6A). CXCL12, a critical factor involved in anoikis and tumor progression [58] was most strongly expressed at the crypt top, forming a clear gradient along the crypt‐surface axis (Fig. S6B,C). These analyses enabled accurate quantification of tumor size and corroborated the increased tumor burden observed in *Tdag8* ^*−/−*^ mice compared with WT controls.

MMP9 is highly expressed in many solid tumors [59], where it contributes to extracellular matrix degradation and tumor invasion. IHC of colon sections revealed significantly increased MMP9 expression in *Tdag8* ^−/−^ mice compared with WT controls (Fig. S7), in line with the larger tumor size observed in this genotype.

Altogether, these results indicate that *Tdag8* deficiency enhances immune activation in intestinal and peripheral compartments. These findings support a protective role of TDAG8 in intestinal homeostasis.

### 3.4 Loss of TDAG8 increases tumor burden in the MC38 *s.c.* injection model

Our results from the AOM/DSS model indicated that TDAG8 plays a protective role against inflammation‐driven colorectal tumorigenesis. To assess whether TDAG8 also influences tumor growth independently of colitis and inflammation, we employed the MC38 *s.c* tumor model, which allows evaluation of the engraftment and progression of mature tumor cells growth in an immune‐competent host without colitis‐associated tissue injury.

**Fig. 4:**
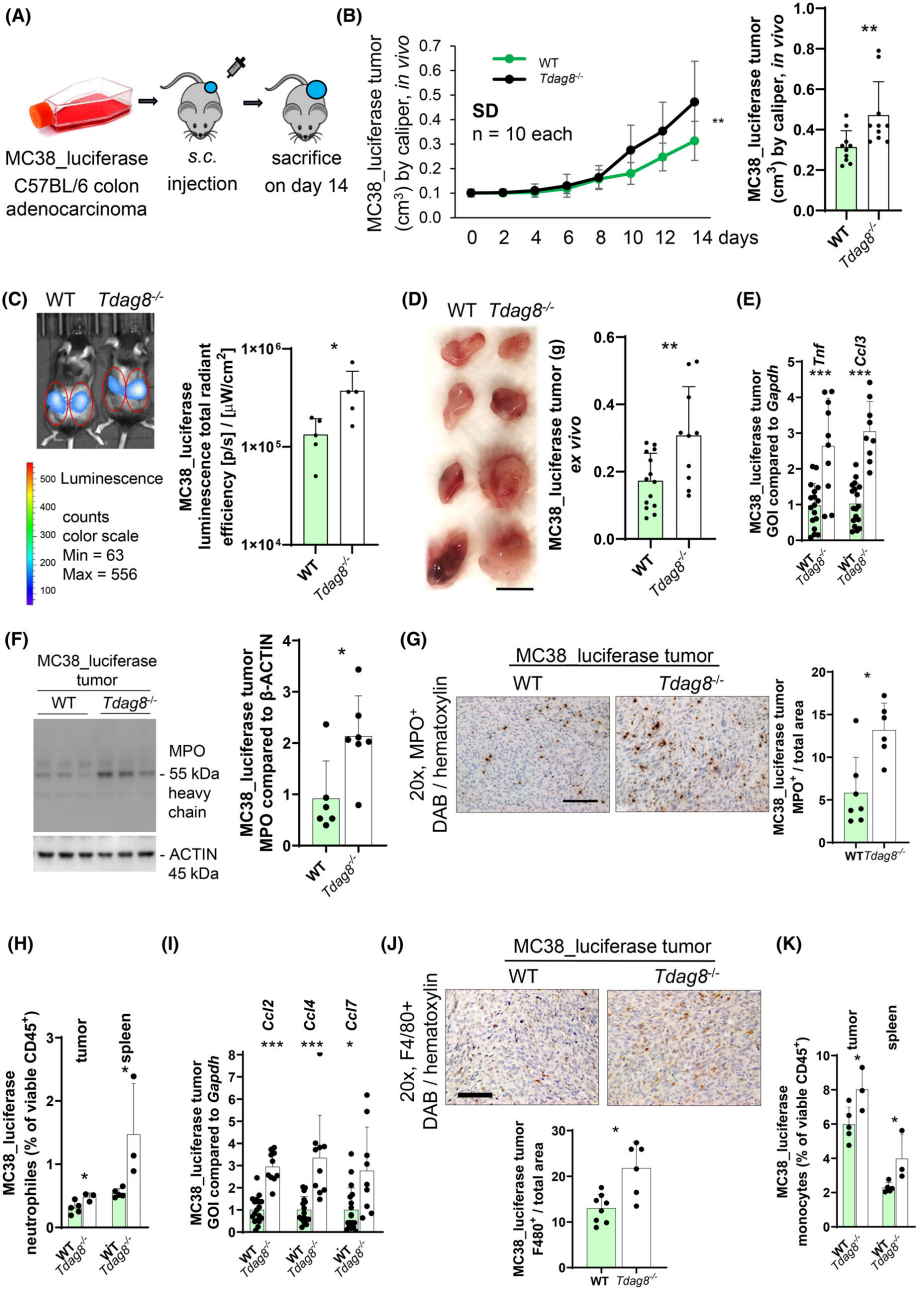
Increased tumor size in *Tdag8* ^−/−^ compared with WT mice in the subcutaneous MC38 model. (A) Tumor induction with MC38 tumor cells. The 300 000 MC38 tumor cells expressing luciferase were injected *s.c*. into WT and *Tdag8* ^−/−^. (B) MC38_luciferase tumor volume over time and quantification (*P* = **, *n* = analysis from 10 tumors from 5 mice for WT and 10 tumors from 5 mice for *Tdag8* ^−−/−^). (C) *In vivo* total radiant efficiency of tumor development on the last day of the experiment and quantification (*P* = *, *n* = analysis from 5 tumors for WT and 5 tumors for *Tdag8* ^−−/−^). (D) Tumor weight and quantification, scale bar 10 mm (*P* = **, *n* = analysis from 14 tumors from 7 mice for WT and 10 tumors from 5 mice for *Tdag8* ^−−/−^). (E) *Tnf* (*P* = ***, *n* = analysis from 17 tumors from 9 mice for WT and 10 tumors from 5 mice for *Tdag8* ^−−/−^) and *Ccl3* qPCR and quantification (*P* = ***, *n* = analysis from 18 tumors from 9 mice for WT and 9 tumors from 5 mice for *Tdag8* ^−−/−^). (F) MPO WB and quantification (*P* = *, n = 6 tumors from 5 mice for WT and 7 tumors from 5 mice for *Tdag8* ^−/−^). (G) MPO IHC and quantification, scale bar 100 μm (DAB brown, hematoxylin blue, *P* = *, *n* = 7 tumors from 5 mice for WT and 6 tumors from 5 mice for *Tdag8* ^−/−^, the statistics include 37 images). (H) Flow cytometry. Neutrophils in tumor tissue (*P* = *, *n* = 5 tumors from 5 mice for WT and 3 tumors from 3 mice for *Tdag8* ^−/−^) and spleen (*P* = *, *n* = 5 spleens for WT and 3 spleens for *Tdag8* ^−/−^) and quantification. (I) qPCR and quantification. *Ccl2* (*P* = ***, *n* = 18 tumors from 9 mice for WT and 10 tumors from 5 mice for *Tdag8* ^−/−^), *Ccl4* (*P* = ***, *n* = 16 tumors from 9 mice for WT and 10 tumors from 5 mice for *Tdag8* ^−/−^) and *Ccl7* (*P* = *, *n* = 18 tumors from 9 mice for WT and 9 tumors from 5 mice for *Tdag8* ^−/−^). (J) IHC F4/80 and quantification (DAB brown, hematoxylin blue, *P* = *, *n* = 8 tumors from 5 mice for WT and 6 tumors from 5 mice for *Tdag8* ^−/−^). (K) Flow cytometry and quantification. Monocytes in tumors (*P* = *, *n* = 5 tumors from 5 mice for WT and 3 tumors from 5 mice for *Tdag8* ^−/−^) and spleen (*P* = * *n* = 5 spleens for WT and 3 spleens for *Tdag8* ^−/−^). (B, F, G, I for Ccl4 and Ccl7) Nonparametric distribution (Shapiro–Wilk test), Mann Whitney test. (C–E, H, I for Ccl2, J and K) Normal distribution (Shapiro–Wilk test), unpaired *t*‐test. Error bars indicate ±SD. WT, wild‐type; *s.c*., subcutaneous; DAB, 3,3′‐diaminobenzidine; MPO, myeloperoxidase; WB, western blot.

MC38‐luciferase cells were injected *s.c*. into *Tdag8* ^−/−^ and WT mice to enable *in vivo* bioluminescence monitoring of tumor progression (Fig. 4A). *Tdag8* ^−/−^ mice developed tumors more rapidly and showed significantly increased tumor volume compared with WT controls (Fig. 4B). Bioluminescence imaging confirmed these findings, showing significantly increased tumor‐associated luminescence in *Tdag8* ^−/−^ mice (Fig. 4C). *Ex vivo* measurements further revealed significantly increased tumor weight in *Tdag8* ^−/−^ mice compared with WT mice (Fig. 4D).

Of note, in line with results from the AOM/DSS studies, ATAD2 expression was detected in MC38‐luciferase tumors from both *Tdag8* ^−/−^ and WT mice, supporting its specific expression in colorectal cancer cells (Fig. S8). To assess inflammatory changes within the TME, we next quantified cytokine expression. Tumors from *Tdag8* ^−/−^ mice exhibited significantly increased *Tnf* and *Ccl3* mRNA levels compared with WT mice (Fig. 4E). TNF is a potent pro‐inflammatory cytokine that promotes leukocyte activation and survival [60], while CCL3 (MIP‐1alpha) drives recruitment of macrophages [61] and neutrophils [62]. The upregulation of these mediators suggests enhanced cell recruitment and activation in *Tdag8* ^−/−^ tumors.

The abundance of neutrophils in tumor tissue was evaluated using western blot, IHC, and flow cytometry. Consistent with the cytokine profile, MPO protein levels (Fig. 4F), the number of MPO^+^ cells (Fig. 4G and Fig. S9A), and the proportion of neutrophils in tumor and spleen (Fig. 4H and Fig. S9B) were all significantly increased in *Tdag8* ^−/−^ mice compared with WT controls.

Expression of phagocyte‐recruiting cytokines *Ccl2, Ccl4*, and *Ccl7* was likewise significantly elevated in tumors from *Tdag8* ^−/−^ mice compared with WT controls (Fig. 4I). Consistent with this cytokine profile, F4/80^+^ macrophages were more abundant (Fig. 4J and Fig. S9C), and the proportion of monocytes in tumor and spleen (Fig. 4K and Fig. S9D) was significantly increased in *Tdag8* ^−/−^ mice compared with WT controls.

In line with the increased CD4^+^/CD8^+^ ratio observed in SILT and spleen of *Tdag8* ^*−/−*^ mice compared with controls (Fig. 2B,C), IHC analysis confirmed a similar increase in CD4^+^/CD8^+^ proportions within MC38‐luciferase tumors from *Tdag8* ^−/−^ compared with WT mice (Fig. S9E).

A second experimental series using unmodified MC38 cells confirmed these results (Fig. S10A). Flow cytometry again demonstrated significantly increased neutrophil and monocyte infiltration in tumors and spleen of *Tdag8* ^*−/−*^ mice (Figs S10B and S11, Table S1 panel 2).

Together, these findings demonstrate that TDAG8 deficiency significantly exacerbates tumor growth and progression of mature tumor cells. The results support and extend the observations from the inflammation‐driven AOM/DSS model, showing that in both systems, loss of TDAG8 increases tumor burden and is associated with enhanced recruitment of tumor‐associated phagocytes.

## 4 Discussion

This study demonstrates spontaneous tumor formation and growth in the AOM/DSS model of colitis, as well as the growth of ectopically implanted MC38 colon carcinoma cells depending on TDAG8 expression in host animals. The subcutaneous MC38 model and the AOM/DSS model represent distinct pathophysiological approaches in CRC research. While the MC38 model functions as a syngeneic transplantation model that allows for rapid tumor growth in a noncolitis‐associated context, the AOM/DSS model accurately recapitulates the clinical progression of colitis‐associated cancer. A critical difference lies in the microenvironment: unlike the subcutaneous MC38 tumor, which grows isolated within the hypodermis, AOM/DSS‐induced tumors develop directly within the colonic mucosa. Consequently, the tumor tissue in the AOM/DSS model is continuously exposed to the intestinal microbiome, which significantly influences tumorigenesis and the local immune response.

Our study demonstrates that spontaneous tumor formation and growth are increased in mice lacking the pH‐sensing receptor TDAG8. In the AOM/DSS model, our results are consistent with those reported by Marie *et al*., who showed that deletion of *Tdag8* is associated with an increase in both tumor number and volume [6].

In *Tdag8* ^*−/−*^ mice, tissue infiltration by macrophages was significantly elevated in the AOM/DSS model compared with WT mice, accompanied by elevated *Tnf* and *Il6* transcript levels. We also observed an increased presence of MMP9‐positive cells in tumor tissue from *Tdag8* ^−/−^ animals following AOM/DSS exposure. Similarly, in the MC38 model, tumor growth was accelerated and tumor tissue showed increased infiltration by monocytes and neutrophils in *Tdag8*‐deficient animals compared with WT.

MMP9 is a key effector molecule secreted by neutrophils and other cell types within the TME. This endopeptidase degrades denatured collagens and basement membranes and serves as a biomarker of intestinal inflammation [63, 64]. It is also considered a potential biomarker for several cancers, with a well‐established role in cancer progression and metastasis [65]. MMP9 inhibition reduces tumor growth rate and weight in an orthotopic xenograft model of CRC, and MMP9 has been proposed as a promising therapeutic target for metastatic CRC [66].

Taken together, our data confirm that the pH‐sensing receptor TDAG8 protects against intestinal inflammation and regulates both tumorigenesis and tumor growth, likely by modulating immune cell infiltration and activity within tumor tissue.

While the role of TDAG8 in controlling inflammation is well established, the outcome of our study regarding tumor development and growth was not necessarily anticipated. On one hand, increased inflammation in TDAG8^−/−^ animals would be expected to promote tumor development in the AOM/DSS model [49, 50]. On the other hand, progression of these tumors, as well as those implanted subcutaneously in the MC38 model, might have been expected to be restrained by enhanced anti‐tumor activity of the TDAG8‐deficient immune system.

Indeed, a study by Bohn *et al*. [67] suggested that acidosis sensing in macrophages via TDAG8 and possibly other Gs‐coupled receptors can lead to tumor immunoevasion in a B16 melanoma model, but not in the MC38 colon carcinoma model. The difference between the two models was attributed to the higher glycolytic activity of B16 compared with MC38 cells. This finding, in line with other observations [36, 68, 69], prompted the development of specific TDAG8 receptor antagonists by Pathios Therapeutics. One such compound increased immune cell infiltration into tumors and reduced subcutaneous MC38 tumor cell growth in mice [70].

At present, the discrepancies between our findings and those reported by Corbin *et al*. remain unresolved. One possibility is that pharmacological inhibition of TDAG8 by an orally administered antagonist does not achieve complete receptor blockade, which may confer a therapeutic advantage compared with the total loss of function observed in knockout models.

## 5 Conclusion

Loss of TDAG8 aggravates intestinal inflammation and promotes colon tumor growth, indicating a protective role for this receptor in colorectal cancer and highlighting TDAG8 as a potential therapeutic target.

## Conflict of interest

EM, YI, AB, MW, RFM, SO, CS, PAR, CdeV, KS, and MH declare no competing interests. GR discloses consulting to Abbvie, Arena, Augurix, BMS, Boehringer, Calypso, Celgene, FALK, Ferring, Fisher, Genentech, Gilead, Janssen, Lilly, MSD, Novartis, Pfizer, Phadia, Roche, UCB, Takeda, Tillots, Vifor, Vital Solutions, and Zeller; speaker's honoraria from Abbvie, Astra Zeneca, BMS, Celgene, FALK, Janssen, MSD, Pfizer, Phadia, Takeda, Tillots, UCB, Vifor, and Zeller; educational grants and research grants from Abbvie, Ardeypharm, Augurix, Calypso, FALK, Flamentera, MSD, Novartis, Pfizer, Roche, Takeda, Tillots, UCB, and Zeller. Gerhard Rogler is cofounder and head of the scientific advisory board of PharmaBiome.

## Authors contributions

EM and AB contributed to the data acquisition (scRNAseq), data curation, writing – review and editing, and final approval. YI contributed to the data acquisition (IHC, qPCR, and quantification of AP sites), data curation, writing – original draft, and writing review and editing. MW and RFM contributed to the data acquisition (IHC), data curation, writing – review and editing. SO contributed to the data acquisition (IHC), data curation, writing – review and editing, and final approval. CS contributed to the data acquisition (IHC and qPCR), data curation, writing – review and editing, and final approval. PAR, CdeV, and KS contributed to the data acquisition (scRNAseq), data curation, writing – review and editing. MH contributed to the data acquisition (animal experiments, IHC, qPCR, flow cytometry, quantification of AP sites, and scRNAseq), data curation, writing – original draft, writing – review and editing, and final approval. GR contributed to the conceptualization, writing – review and editing. All authors approved the final submitted version of the manuscript.

## Supporting information


**Fig. S1.** *TDAG8* is predominantly expressed in T cells.
**Fig. S2.** Increased inflammation in *Tdag8* ^−/−^ compared with WT mice upon AOM/DSS colitis.
**Fig. S3.** Increased number of macrophages in *Tdag8* ^−/−^ compared with WT mice upon AOM/DSS colitis.
**Fig. S4.** Signs of tumor development in progress colonoscopy in *Tdag8* ^−/−^ compared with WT mice upon AOM/DSS.
**Fig. S5.** ATAD2 in *Tdag8* ^−/−^ compared with WT mice.
**Fig. S6.** Identification of tumors by IHC.
**Fig. S7.** Increased MMP9 in *Tdag8* ^−/−^ compared with WT mice upon AOM/DSS colitis.
**Fig. S8.** Number of ATAD2 ^+^ cells remain constant in *Tdag8* ^−/−^ compared with WT mice in tumors from the MC38 model.
**Fig. S9.** Increased number of CD4^+^/CD8^+^ ratio in *Tdag8* ^−/−^ compared with WT mice.
**Fig. S10.** Increased number of monocytes in *Tdag8* ^−/−^ compared with WT mice.
**Fig. S11.** Increased number of monocytes in *Tdag8* ^−/−^ compared with WT mice.

**Table S1.** Antibody panels used for flow cytometry.

**Data S1.** Supplementary Legends.

## Data Availability

The data underlying this article and supporting data are available as Supplementary Information and in a public database: https://doi.org/10.6084/m9.figshare.32167623.
