# Supplementary material for: Loss of proton‐sensing TDAG8 increases tumor progression in mouse models of colon cancer
Source: Mol Oncol. 2026 Jun 9;20(11):2656–73. doi: 10.1002/1878-0261.70283 (PMC13398714; doi:10.1002/1878-0261.70283)
Supplement: Supplementary file 1 [file MOL2-20-2656-s001.docx]

**SUPPLEMENTARY FIGURE LEGENDS**

**Supplementary Figure 1: *TDAG8* is predominantly expressed in T cells.** Murine CRC analysis. **(A)** UMAP plot of murine colonic tumor tissue showing distinct cell populations. **(B)** Heatmap showing differentially expressed genes across all identified clusters. **(C)** Feature plots displaying the expression patterns of selected markers of early colorectal tumorigenesis (*Notum* and *Tacstd2)* across the UMAP embedding.

**Supplementary Figure 2: Increased inflammation in *Tdag8*^-/-^ compared with WT mice upon AOM/DSS colitis. (A)** Spleen weight, quantification (left, p = 0.4084, n= 6 mice for WT and 5 mice for *Tdag8*^-/-^) and ratio of spleen weight to body weight (right, p = 0.6935). **(B)** Histological score, colon, and quantification. Scale bar for panorama 500 µm and for 20x 100 µm (p = 0.3021 for epithelial damage and p = 0.0757 for lymphocyte influx, n= 11 mice for WT and 4 mice for *Tdag8*^-/-^). **(C)** Histological score, small bowel, and quantification. Scale bar for panorama 500 µm and for 20x 100 µm (p = 0.8484 for epithelial damage and p = 0.2171 for lymphocyte influx, n= 12 mice for WT and 4 mice for *Tdag8*^-/-^). **(A and B)** Normal distribution (Shapiro-Wilk test), unpaired t-test. ±SD. **(C)** Non-parametric distribution (Shapiro-Wilk test), unpaired t-test. ±SD. WT, wild-type; DAB, 3,3’-diaminobenzidine; AOM, azoxymethane; DSS, dextran sodium sulfate.

**Supplementary Figure 3: Increased number of macrophages in *Tdag8*^-/-^ compared with WT mice upon AOM/DSS colitis. (A)** Dot Plots showing expression for *IL6*, *CCL3*, and *TNF* in single cell annotation datasets from human (left) and mouse (right). **(B)** IHC, F4/80, macrophages. Scale bar for 20x 100 µm (DAB brown, hematoxylin blue, representative images for n = 3 mice for WT and 3 mice for *Tdag8*^-/-^). WT, wild-type; DAB, 3,3’-diaminobenzidine.

**Supplementary Figure 4: Signs of tumor development in progress colonoscopy in *Tdag8*^-/-^ compared with WT mice upon AOM/DSS. (A)** Progress colonoscopy. **(B)** Colon length and quantification. Macroscopic mucosal view of the colon before (left) and after (right) longitudinal incision. Images taken with a smartphone. Scale bars 5mm (n = 6 mice for WT and 5 mice for *Tdag8*^-/-^). Normal distribution (Shapiro-Wilk test), unpaired t-test. ±SD. WT, wild-type.

**Supplementary Figure 5: ATAD2 in *Tdag8*^-/-^ compared with WT mice.** Tumor induction with AOM/DSS. Paraffin sections of mice colon. **(A)** STMN1. 20x. Scale bar 100 µm. **(B)** IHC and quantification, ATAD2^+^ cells. 20x. Scale bar 100 µm (p = 0.3763, n = 8 mice for WT and 5 mice for *Tdag8*^-/-^). **(C)** STMN1, detail. Original magnification 20x. Scale bar 100 µm. Normal distribution (Shapiro-Wilk test), unpaired t-test. ±SD. DAB brown, hematoxylin blue, dashed line = tumor, representative images. t = tumor. WT, wild-type; DAB, 3,3’-diaminobenzidine.

**Supplementary Figure 6: Identification of tumors by IHC. (A)** CK20. Scale bar for 10x as indicated and scale bar for detail 50 µm (representative images). CXCL12 in **(B)** crypts, Detail, original magnification 20x and **(C)** tumor tissue, 20x. Scale bar 100 µm (DAB brown, hematoxylin blue, dashed line = tumor, representative images). c = colonic crypt, t = tumor. WT, wild-type; DAB, 3,3’-diaminobenzidine.

**Supplementary Figure 7: Increased MMP9 in *Tdag8*^-/-^ compared with WT mice upon AOM/DSS colitis.** IHC for MMP9, scale bar 100 µm (DAB brown, hematoxylin blue, p = *, n = analysis from 12 images from 4 mice for WT and 9 images from 3 mice for *Tdag8*^--/-^). Normal distribution (Shapiro-Wilk test), unpaired t-test. ±SD. WT, wild-type; DAB, 3,3’-diaminobenzidine; AOM, azoxymethane; DSS, dextran sodium sulfate.

**Supplementary Figure 8: Number of ATAD2 ^+^ cells remain constant in *Tdag8*^-/-^ compared with WT mice in tumors from the MC38 model.** 300,000 MC38 tumor cells expressing luciferase were injected *s.c.* into WT and *Tdag8*^-/-^. IHC, ATAD2, and quantification. Scale bar for 20x 100 µm. (DAB brown, hematoxylin blue, p = 0.9730, n= 8 tumors for WT and 6 tumors for *Tdag8*^-/-^). Normal distribution (Shapiro-Wilk test), unpaired t-test. ±SD. WT, wild-type; *s.c.*, subcutaneous; DAB, 3,3’-diaminobenzidine.

**Supplementary Figure 9:** **Increased number of CD4^+^/CD8^+^ ratio in *Tdag8*^-/-^ compared with WT mice.** Tumor induction with MC38 tumor cells. **A)** MPO IHC, scale bar 100 µm, pseudo color and area for quantification. IHC in thymus for positive and negative control (DAB brown, hematoxylin blue). **(B)** Flow cytometry, waterfall for neutrophiles in tumor tissue and spleen. **(C)** IHC F4/80, scale bar 100 µm, pseudo color and area for quantification. IHC in thymus for positive and negative control (DAB brown, hematoxylin blue). **(D)** Flow cytometry, waterfall for monocytes in tumors and spleen. **(E)** IHC in MC38 tumors and quantification, CD4^+^ and CD8^+^ T cells. Scale bar for 20x 100 µm (DAB brown, hematoxylin blue, p = 0,0217, n = 6 mice for WT and 6 mice for *Tdag8*^-/-^, error bars indicate ±SD). IHC in thymus for positive and negative control. WT, wild-type; DAB, 3,3’-diaminobenzidine; MPO, myeloperoxidase.

**Supplementary Figure 10: Increased number of monocytes in *Tdag8*^-/-^ compared with WT mice.** Tumor induction with MC38 tumor cells. **(A)** 300,000 standard MC38 without luciferase were injected *s.c.* into WT and *Tdag8*^-/-^. **(B)** Flow cytometry. Neutrophiles in tumor tissue or spleen and quantification (p = *, n= 5 tumors for WT and 3 tumors for *Tdag8*^-/-^). Normal distribution (Shapiro-Wilk test), unpaired t-test. ±SD. WT, wild-type; *s.c.*, subcutaneous; MPO, myeloperoxidase.

**Supplementary Figure 11: Increased number of monocytes in *Tdag8*^-/-^ compared with WT mice.** Tumor induction with MC38 tumor cells. 300,000 standard MC38 without luciferase were injected *s.c.* into WT and *Tdag8*^-/-^. Flow cytometry. Monocytes in tumor tissue or spleen and quantification (p = *, n= 5 tumors for WT and 3 tumors for *Tdag8*^-/-^). Normal distribution (Shapiro-Wilk test), unpaired t-test. ±SD. WT, wild-type.

**SUPPLEMENTARY TABLE S1**

**Antibody panels used for flow cytometry.** Antibodies used for FACS (M = mouse; R = rat; H = hamster; NA = not applicable).
