# Supplementary material for: Loss of proton‐sensing TDAG8 increases tumor progression in mouse models of colon cancer
Source: Mol Oncol. 2026 Jun 9:10.1002/1878-0261.70283. Online ahead of print. doi: 10.1002/1878-0261.70283 (PMC13398714; doi:10.1002/1878-0261.70283)
Supplement: Supplementary file 1 — Fig. S1. TDAG8 is predominantly expressed in T cells. Fig. S2. Increased inflammation in Tdag8 −/− compared with WT mice upon AOM/DSS colitis. Fig. S3. Increased number of macrophages in Tdag8 −/− compared with WT mice upon AOM/DSS colitis. Fig. S4. Signs of tumor development in progress colonoscopy in Tdag8 −/− compared with WT mice upon AOM/DSS. Fig. S5. ATAD2 in Tdag8 −/− compared with WT mice. Fig. S6. Identification of tumors by IHC. Fig. S7. Increased MMP9 in Tdag8 −/− compared with WT mice upon AOM/DSS colitis. Fig. S8. Number of ATAD2 + cells remain constant in Tdag8 −/− compared with WT mice in tumors from the MC38 model. Fig. S9. Increased number of CD4+/CD8+ ratio in Tdag8 −/− compared with WT mice. Fig. S10. Increased number of monocytes in Tdag8 −/− compared with WT mice. Fig. S11. Increased number of monocytes in Tdag8 −/− compared with WT mice. [file MOL2-9999-0-s003.pptx]

## Slide 1
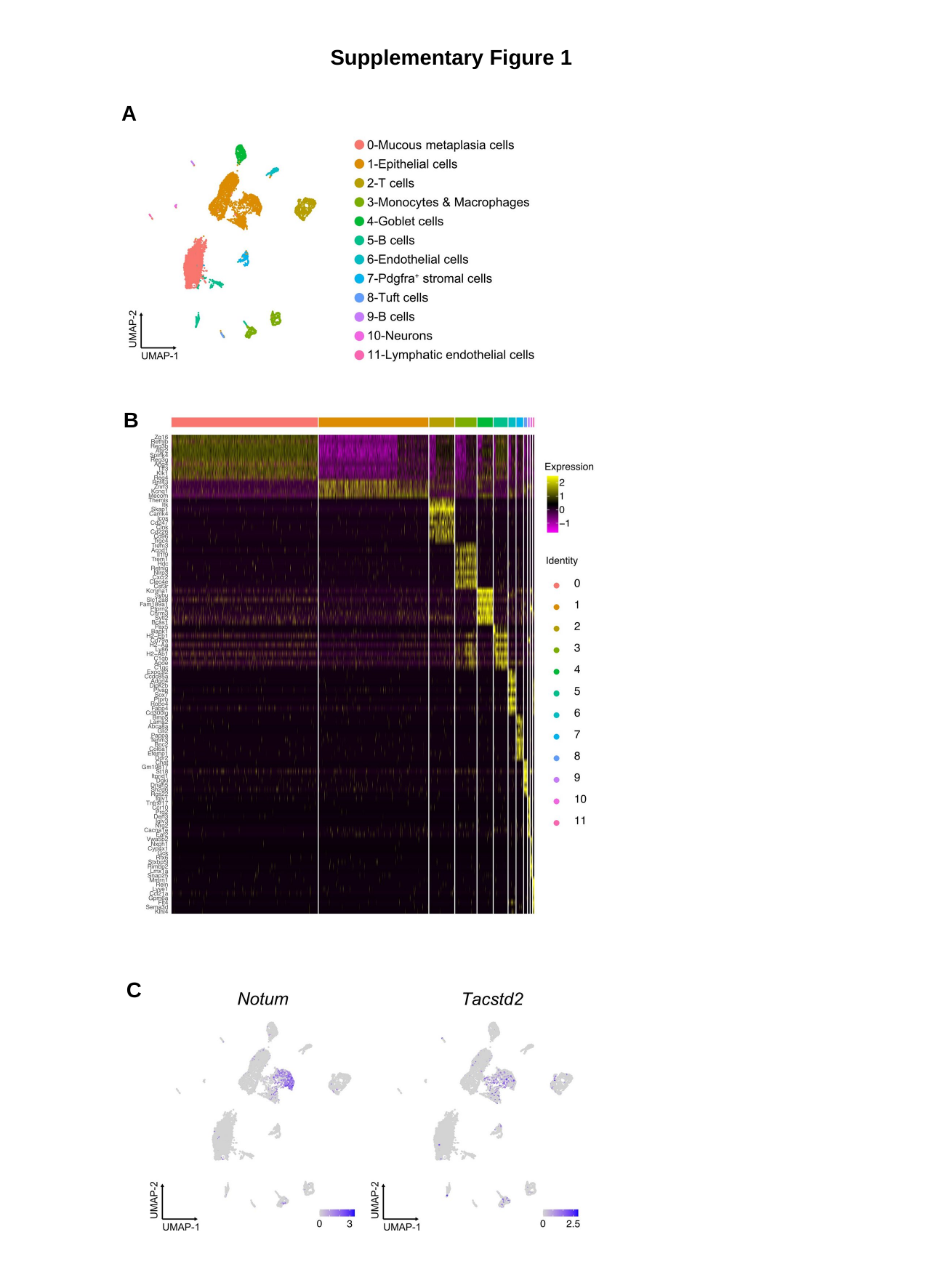

Supplementary Figure 1
A
B
C

## Slide 2
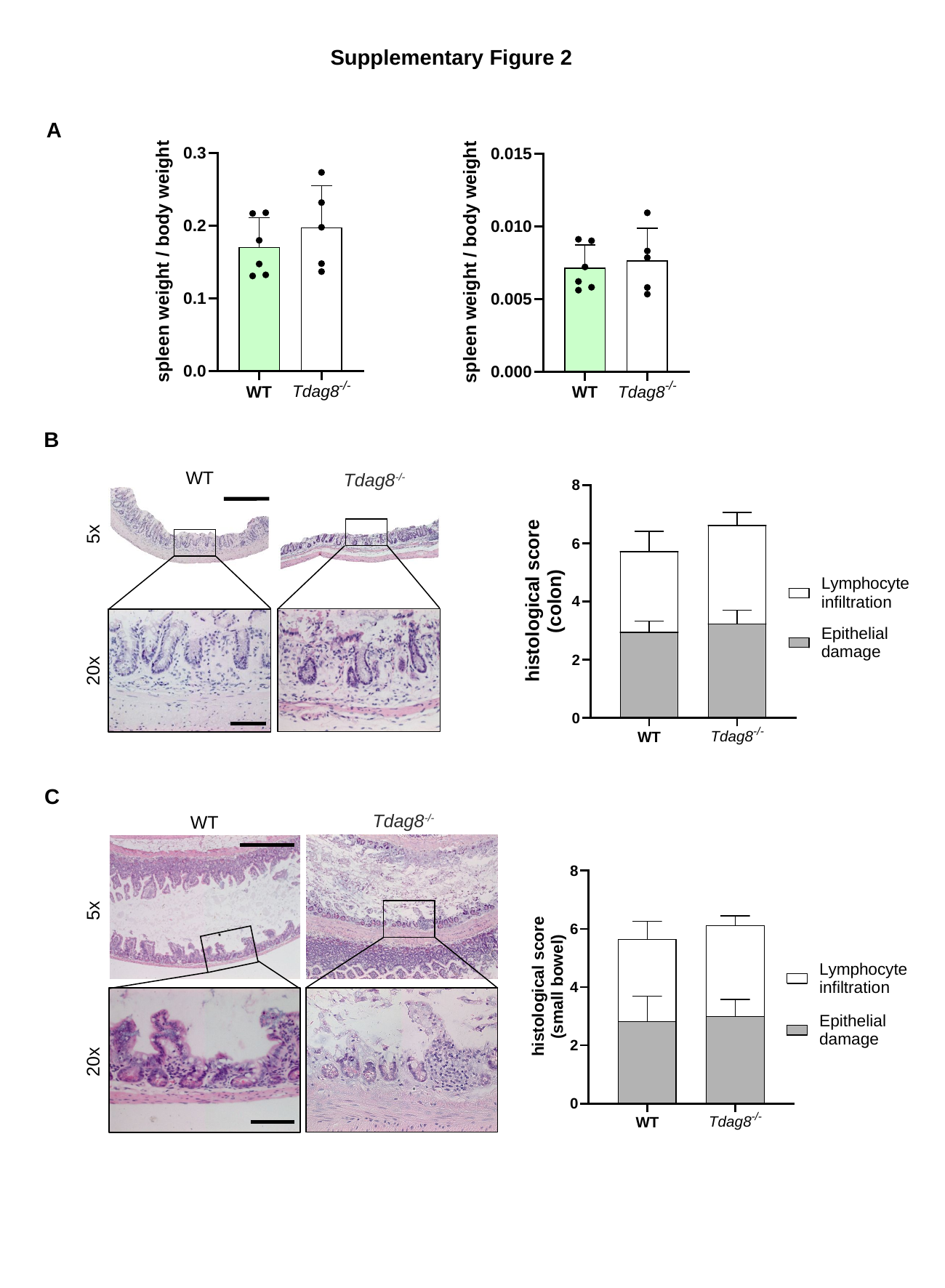

Supplementary Figure 2
A
B
WT
Tdag8-/-
20x 5x
C
Tdag8-/-
WT
20x 5x

## Slide 3
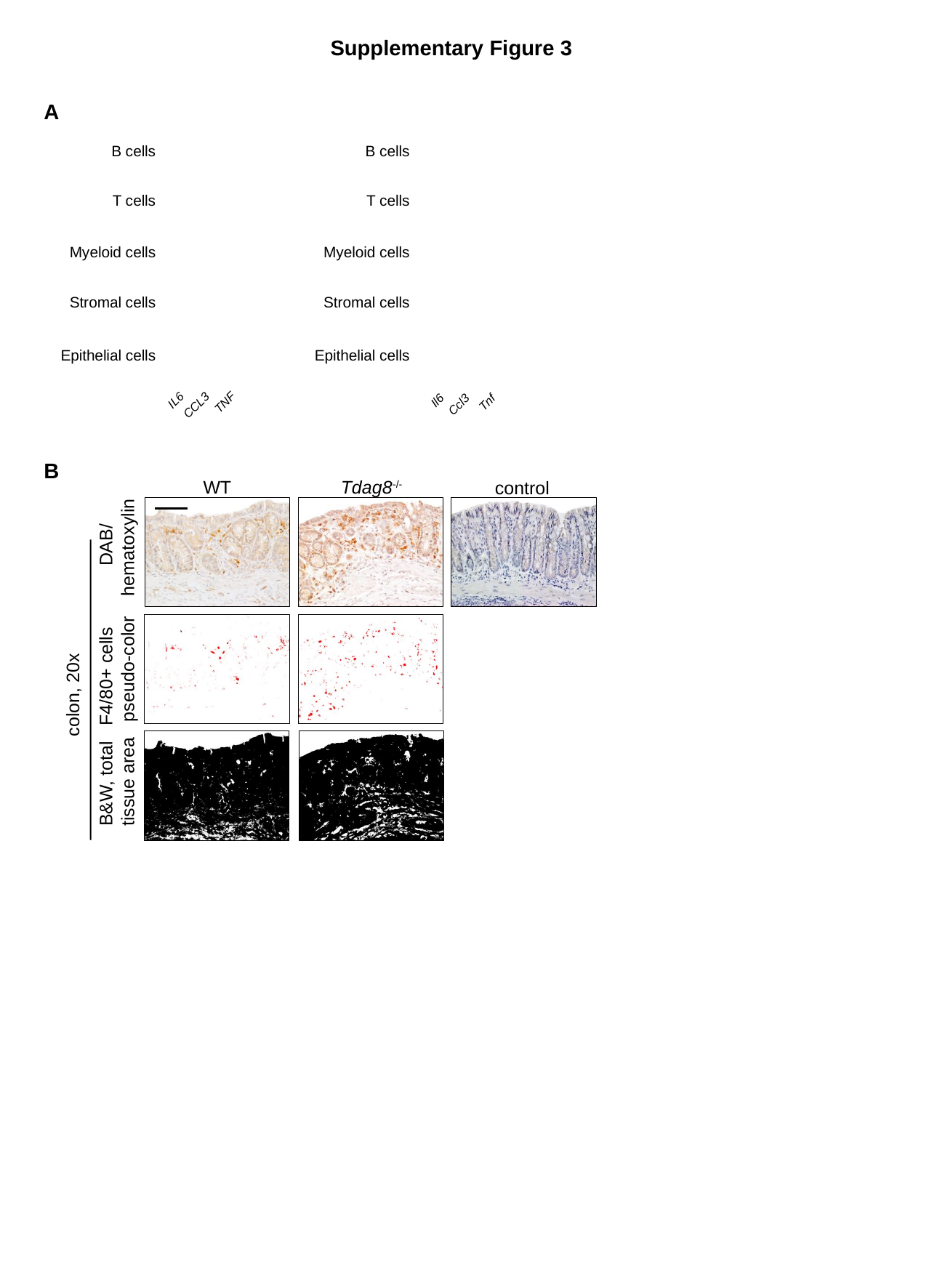

Supplementary Figure 3
A
B cells
B cells
T cells
T cells
Myeloid cells
Myeloid cells
Stromal cells
Stromal cells
Epithelial cells
Epithelial cells
IL6
Il6
CCL3
TNF
Ccl3
Tnf
B
Tdag8-/-
WT
control
B&W, total F4/80+ cells DAB/
tissue area pseudo-color hematoxylin
colon, 20x

## Slide 4
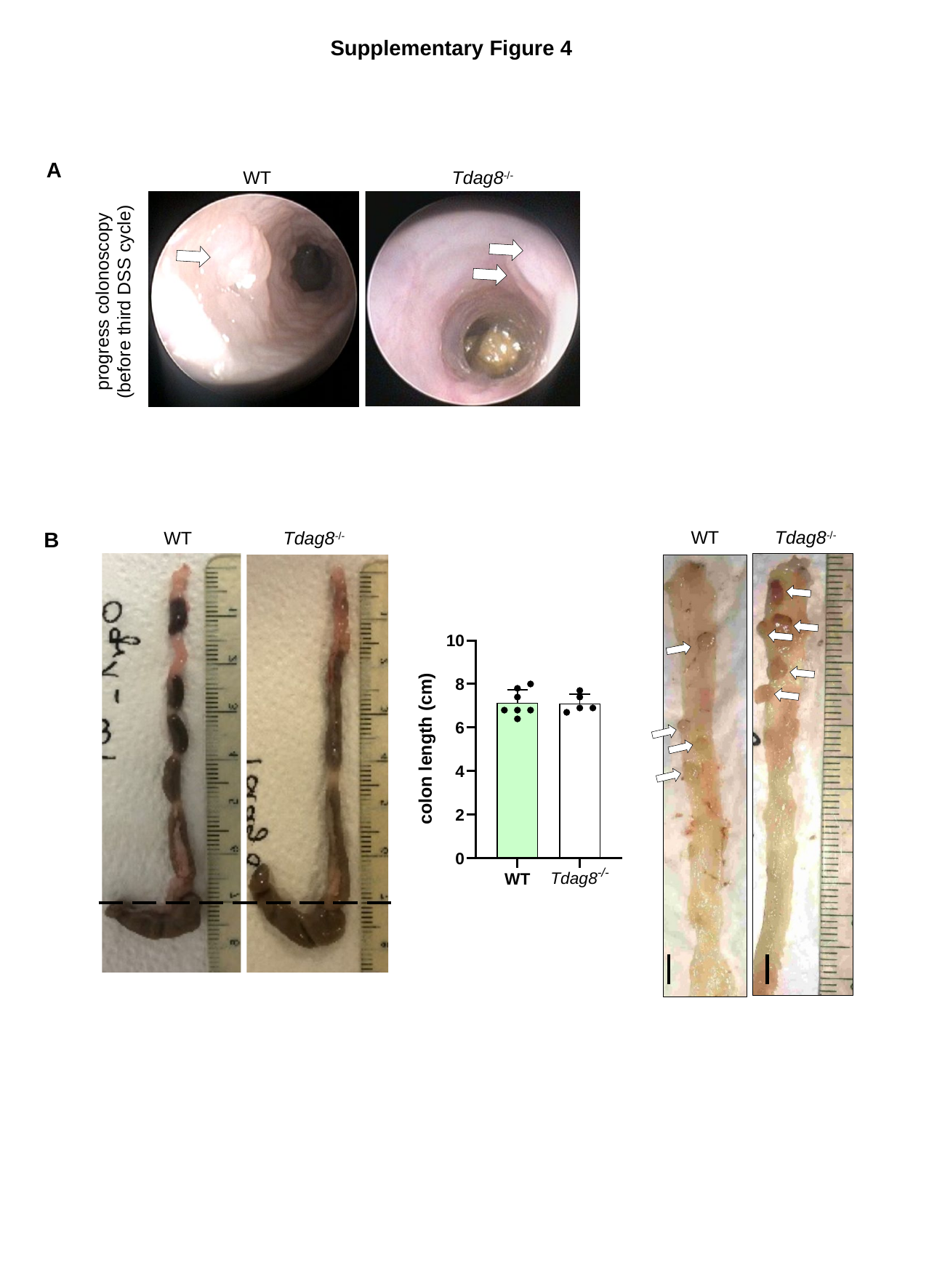

Supplementary Figure 4
A
 WT Tdag8-/-
progress colonoscopy
(before third DSS cycle)
 WT Tdag8-/-
 WT Tdag8-/-
B

## Slide 5
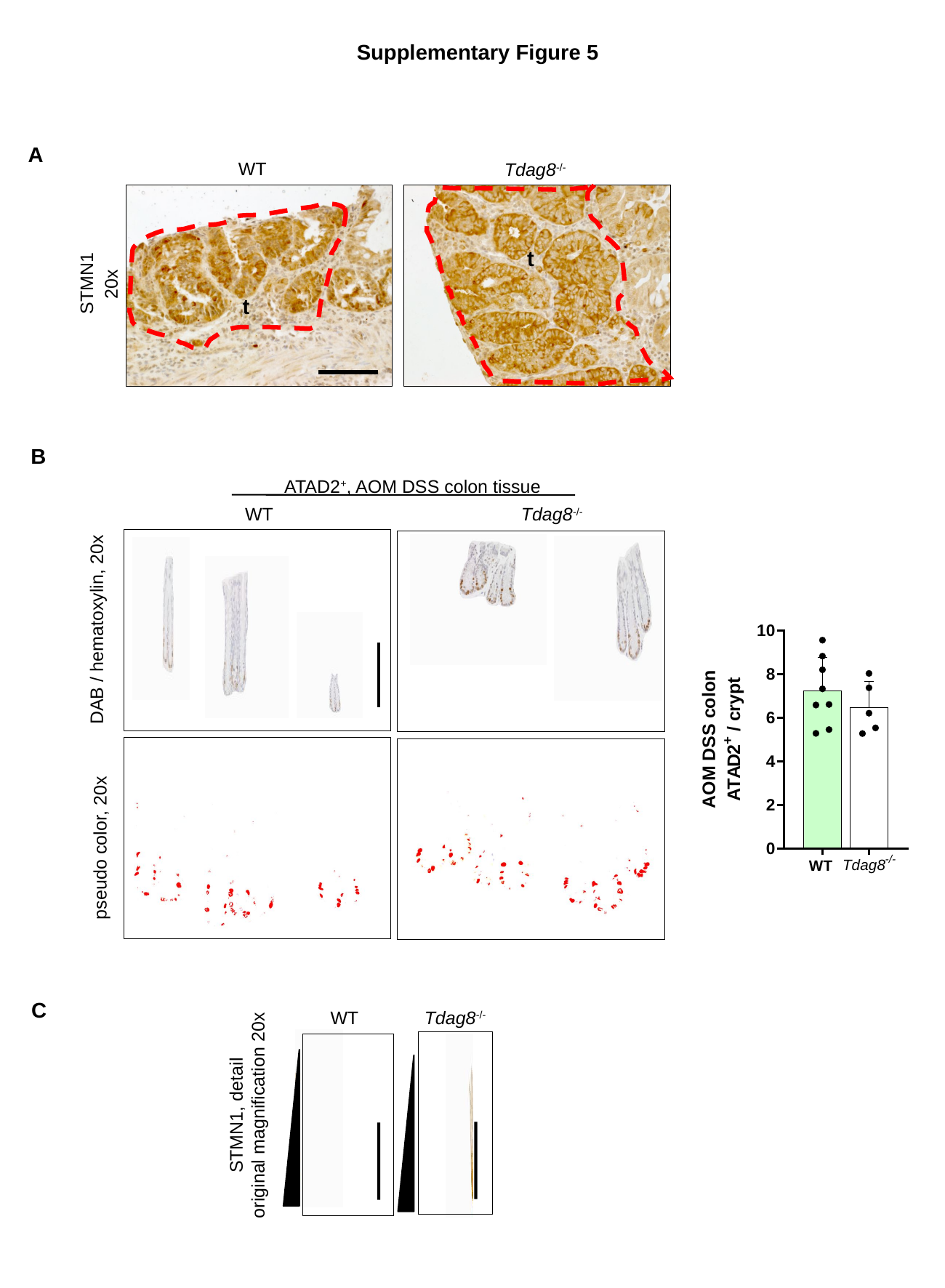

Supplementary Figure 5
A
WT
Tdag8-/-
20x
t
STMN1
t
B
ATAD2+, AOM DSS colon tissue
Tdag8-/-
WT
DAB / hematoxylin, 20x
pseudo color, 20x
C
 WT Tdag8-/-
STMN1, detail
original magnification 20x

## Slide 6
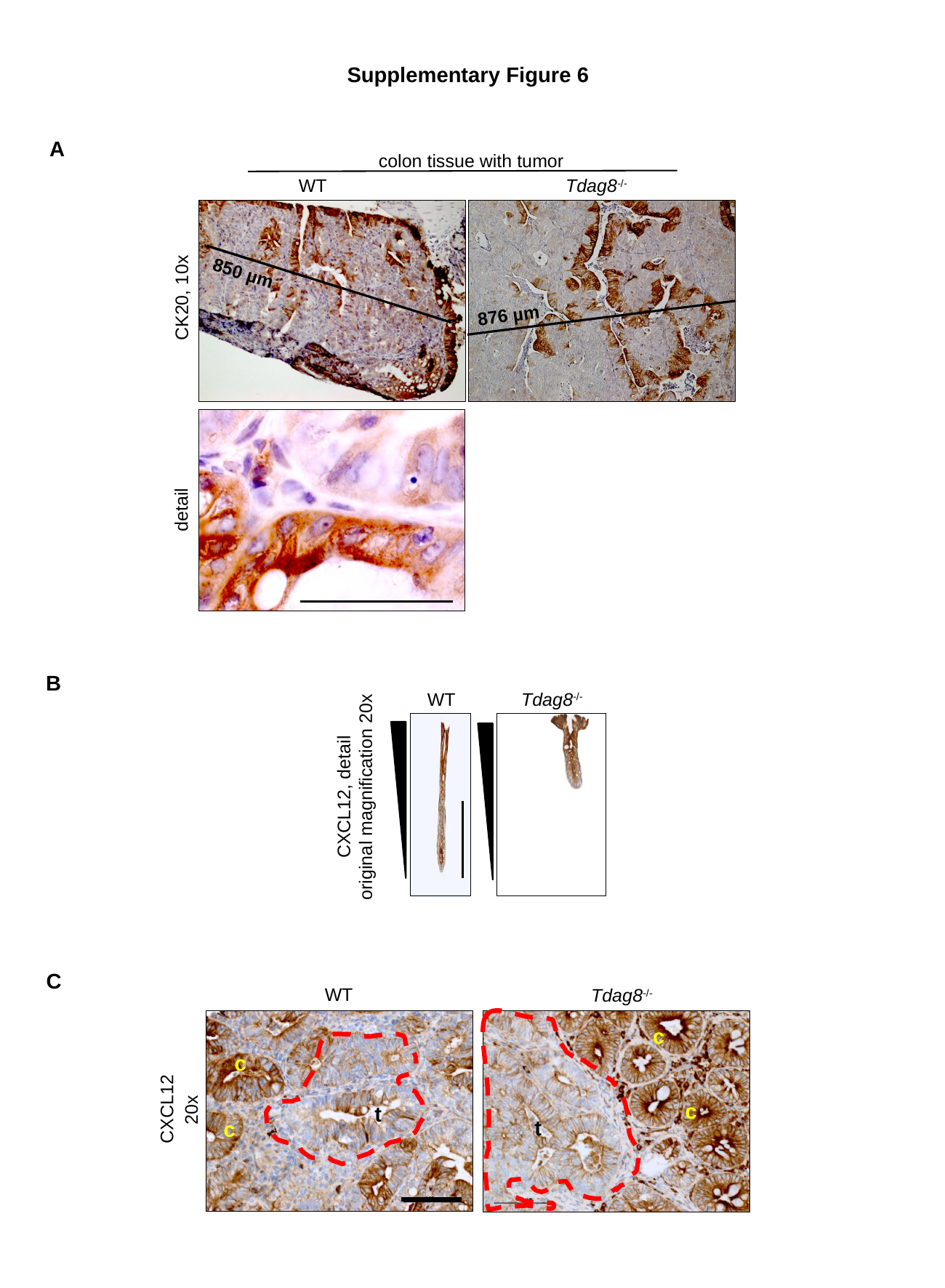

Supplementary Figure 6
A
 colon tissue with tumor
WT Tdag8-/-
850 µm
CK20, 10x
876 µm
detail
B
 WT Tdag8-/-
CXCL12, detail
original magnification 20x
C
WT
Tdag8-/-
c
c
t
c
20x
CXCL12
t
c

## Slide 7
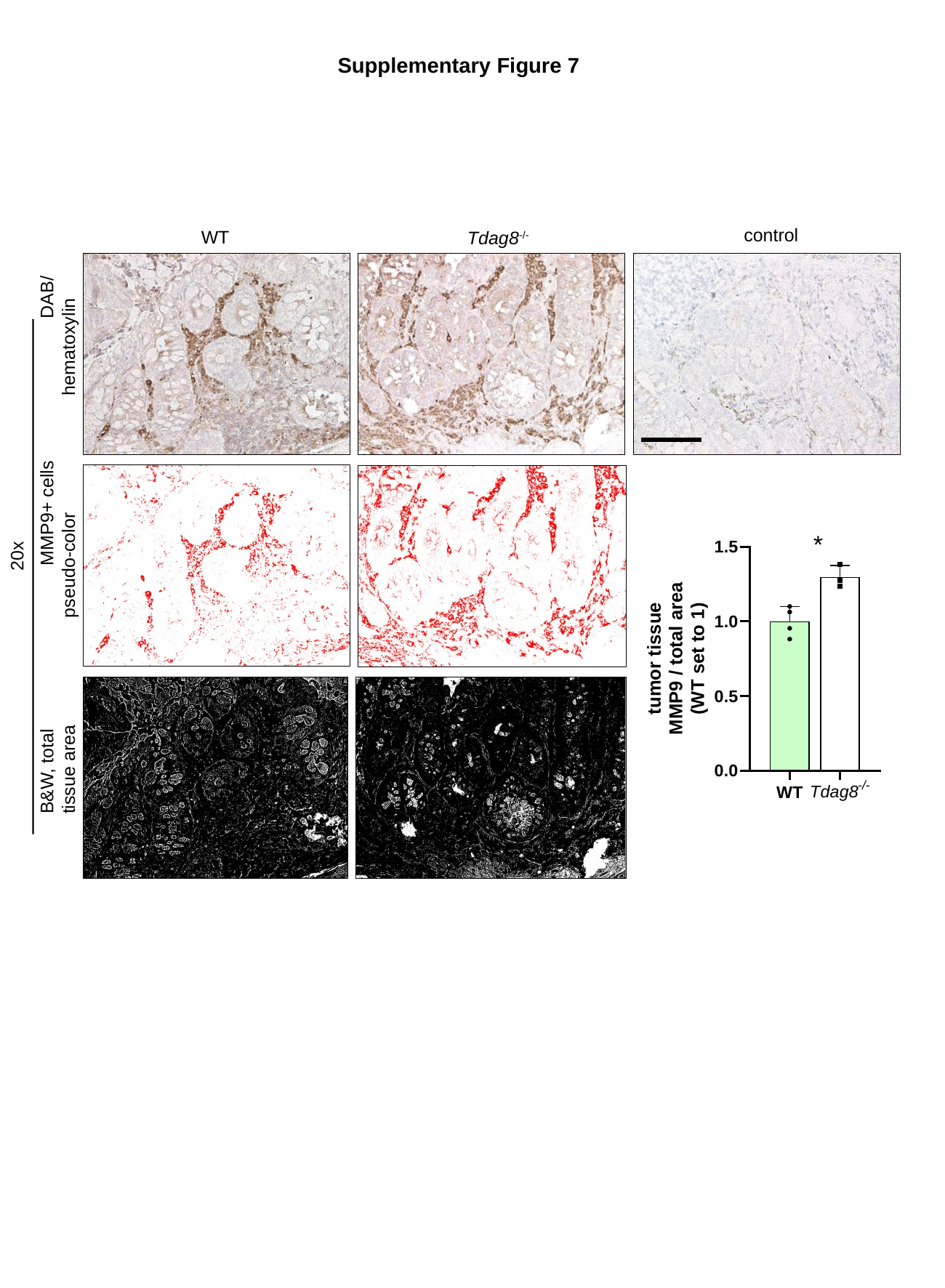

Supplementary Figure 7
control
WT
Tdag8-/-
 B&W, total 	 MMP9+ cells DAB/
 tissue area pseudo-color hematoxylin
20x

## Slide 8
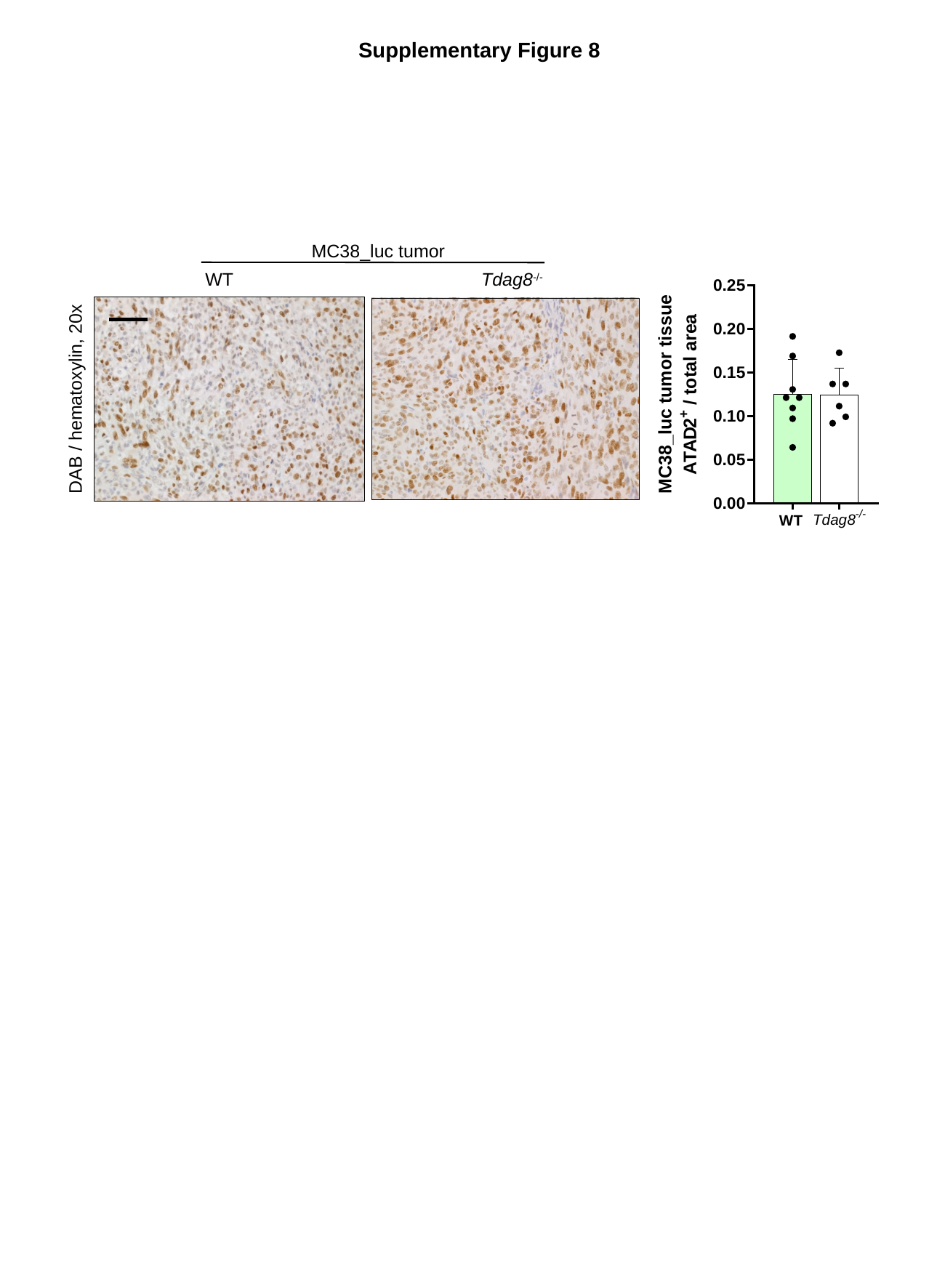

Supplementary Figure 8
MC38_luc tumor
Tdag8-/-
WT
DAB / hematoxylin, 20x

## Slide 9
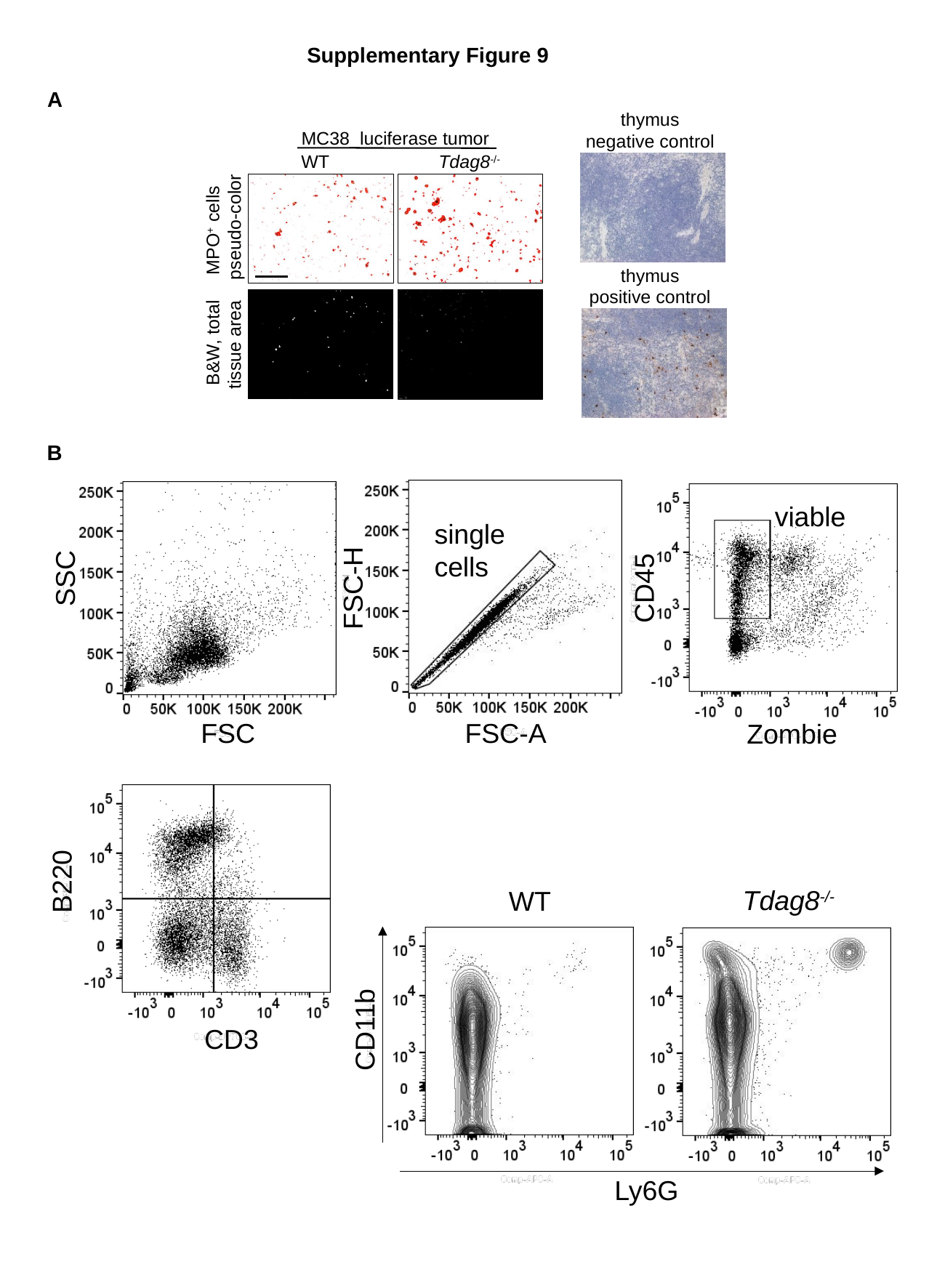

Supplementary Figure 9
A
thymus
negative control
MC38_luciferase tumor
Tdag8-/-
WT
200um
 B&W, total MPO+ cells
 tissue area pseudo-color
thymus
positive control
B
viable
single cells
SSC
CD45
FSC-H
FSC-A
FSC
Zombie
B220
Tdag8-/-
WT
CD11b
CD3
Ly6G

## Slide 10
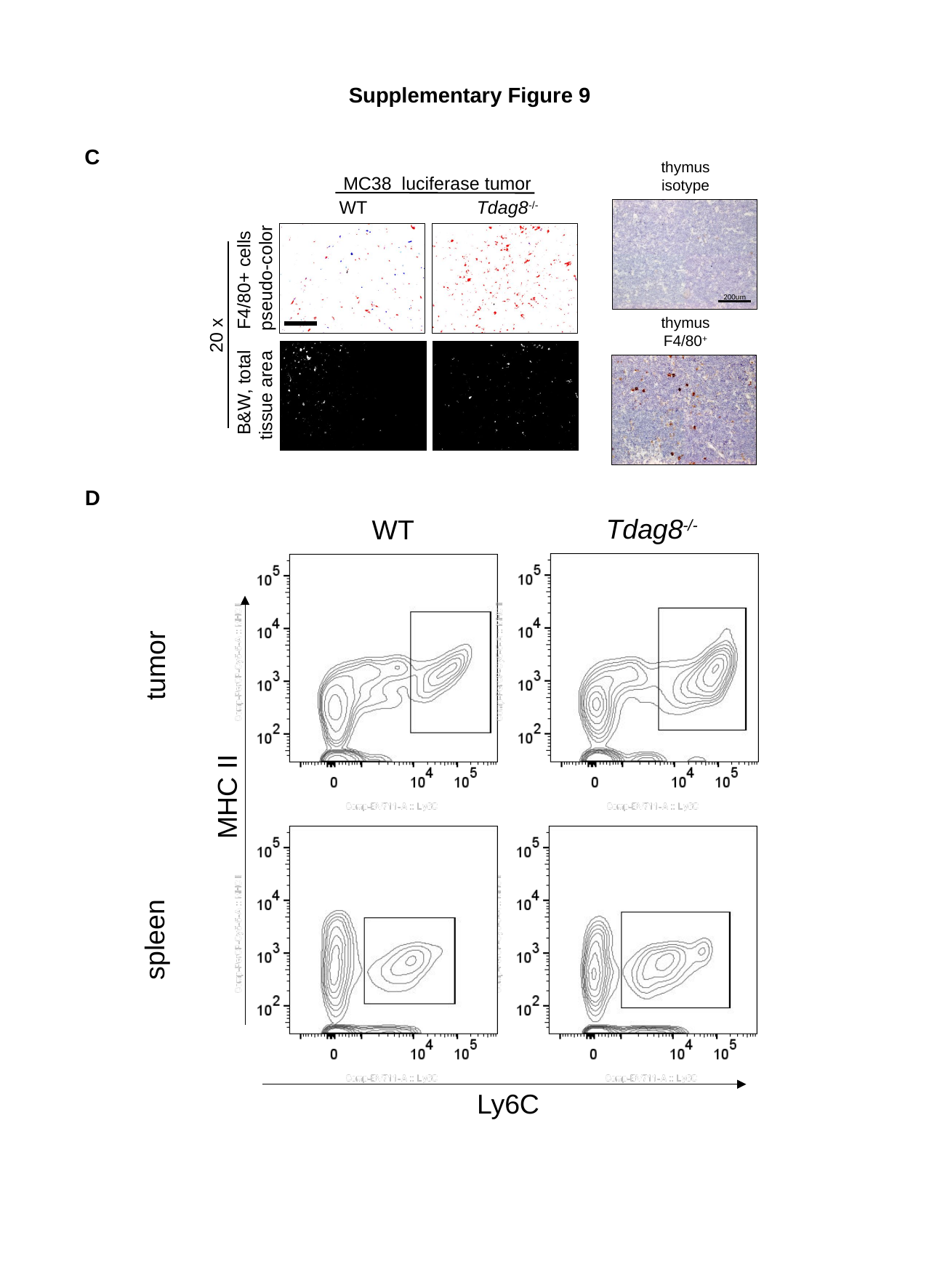

Supplementary Figure 9
C
thymus
isotype
MC38_luciferase tumor
Tdag8-/-
WT
200um
 B&W, total F4/80+ cells
 tissue area pseudo-color
thymus
F4/80+
20 x
D
Tdag8-/-
WT
tumor
MHC II
spleen
Ly6C

## Slide 11
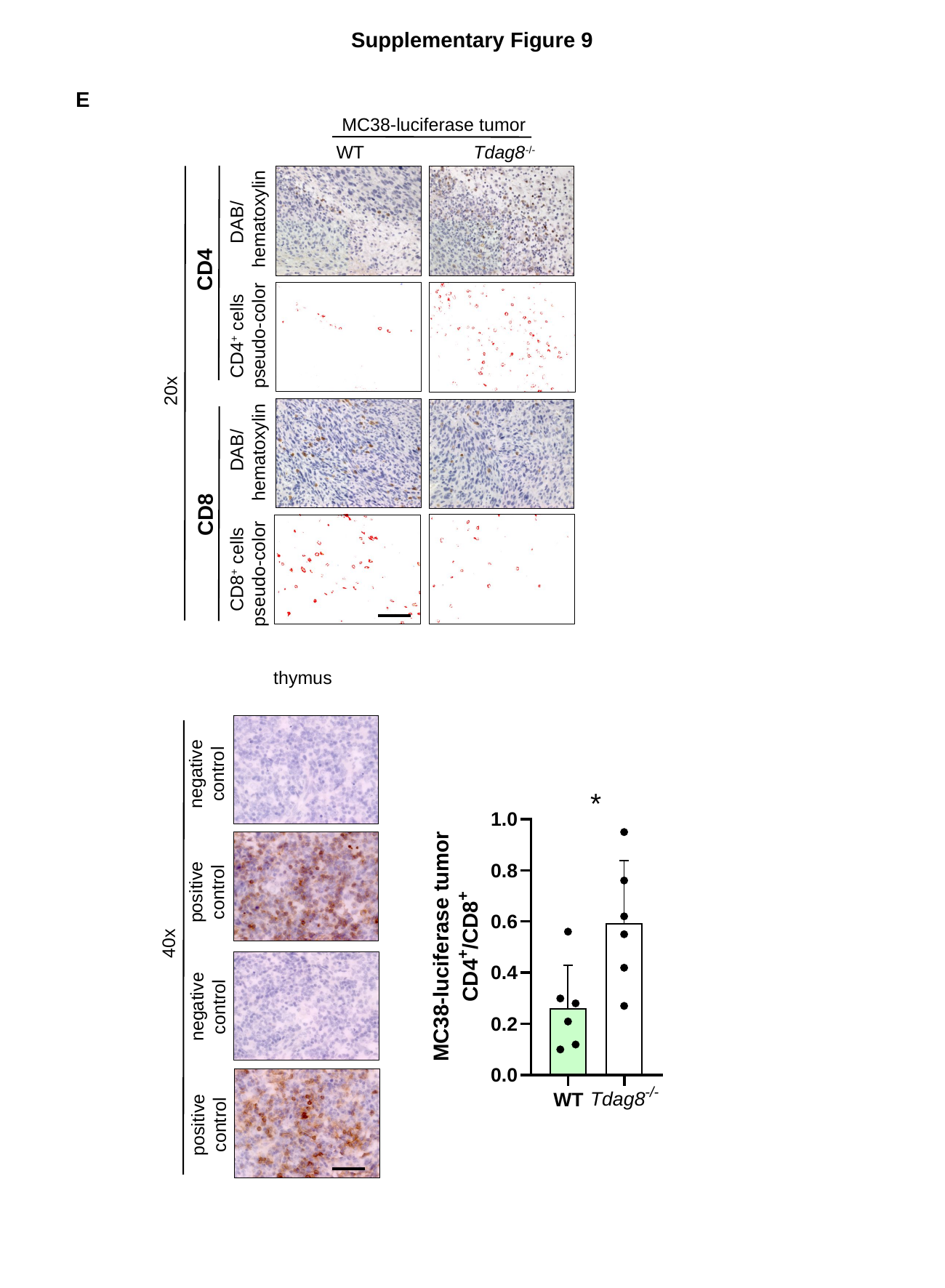

Supplementary Figure 9
E
MC38-luciferase tumor
Tdag8-/-
WT
200 µm
200 µm
CD4
200 µm
200 µm
 CD8+ cells DAB/ 	 CD4+ cells DAB/ pseudo-color hematoxylin pseudo-color hematoxylin
20x
CD8
thymus
negative control
positive control
40x
negative control
positive control

## Slide 12
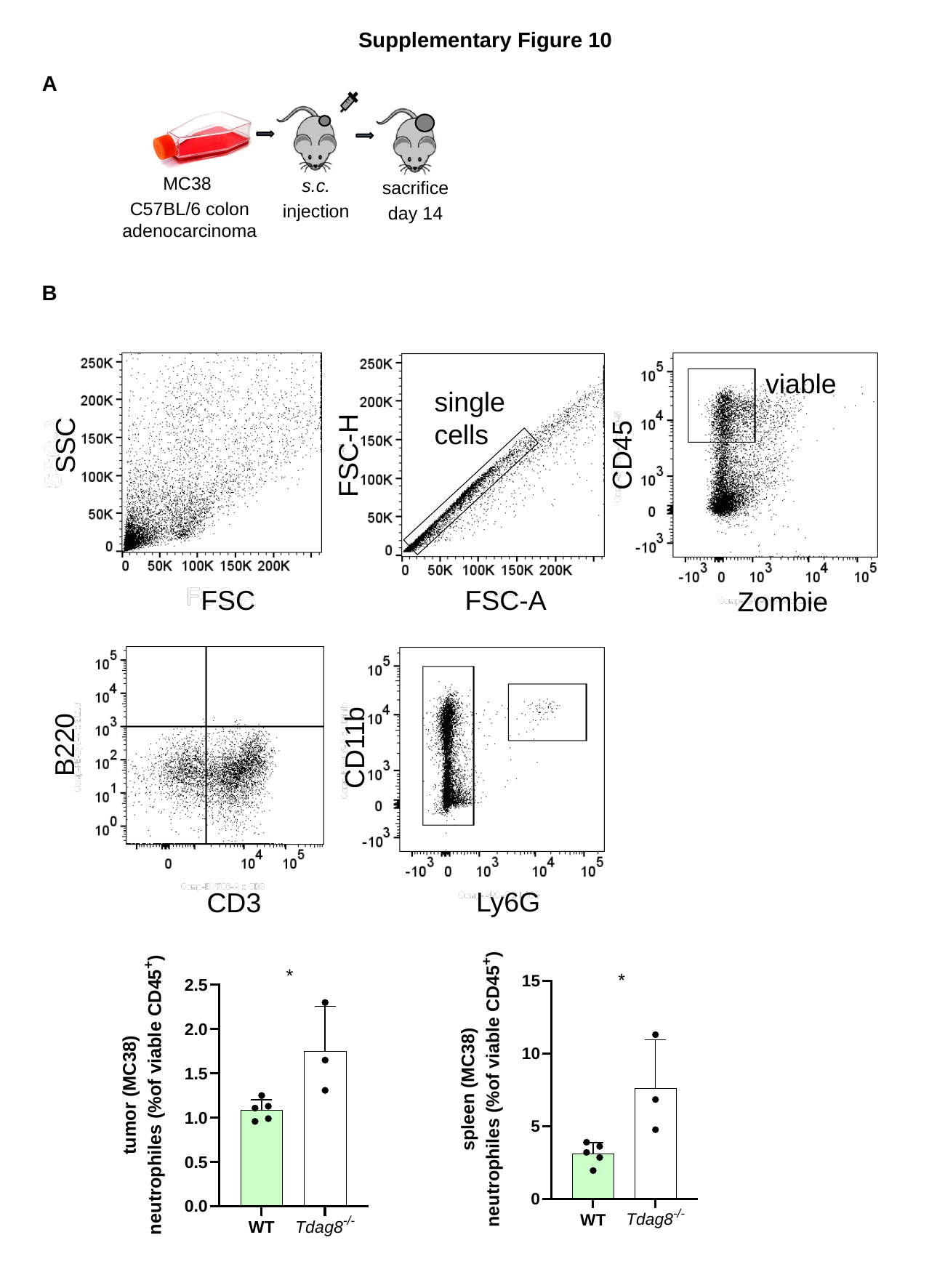

Supplementary Figure 10
A
MC38
C57BL/6 colon adenocarcinoma
s.c.
injection
sacrifice
day 14
B
viable
single cells
SSC
CD45
FSC-H
FSC-A
FSC
Zombie
CD11b
B220
Ly6G
CD3

## Slide 13
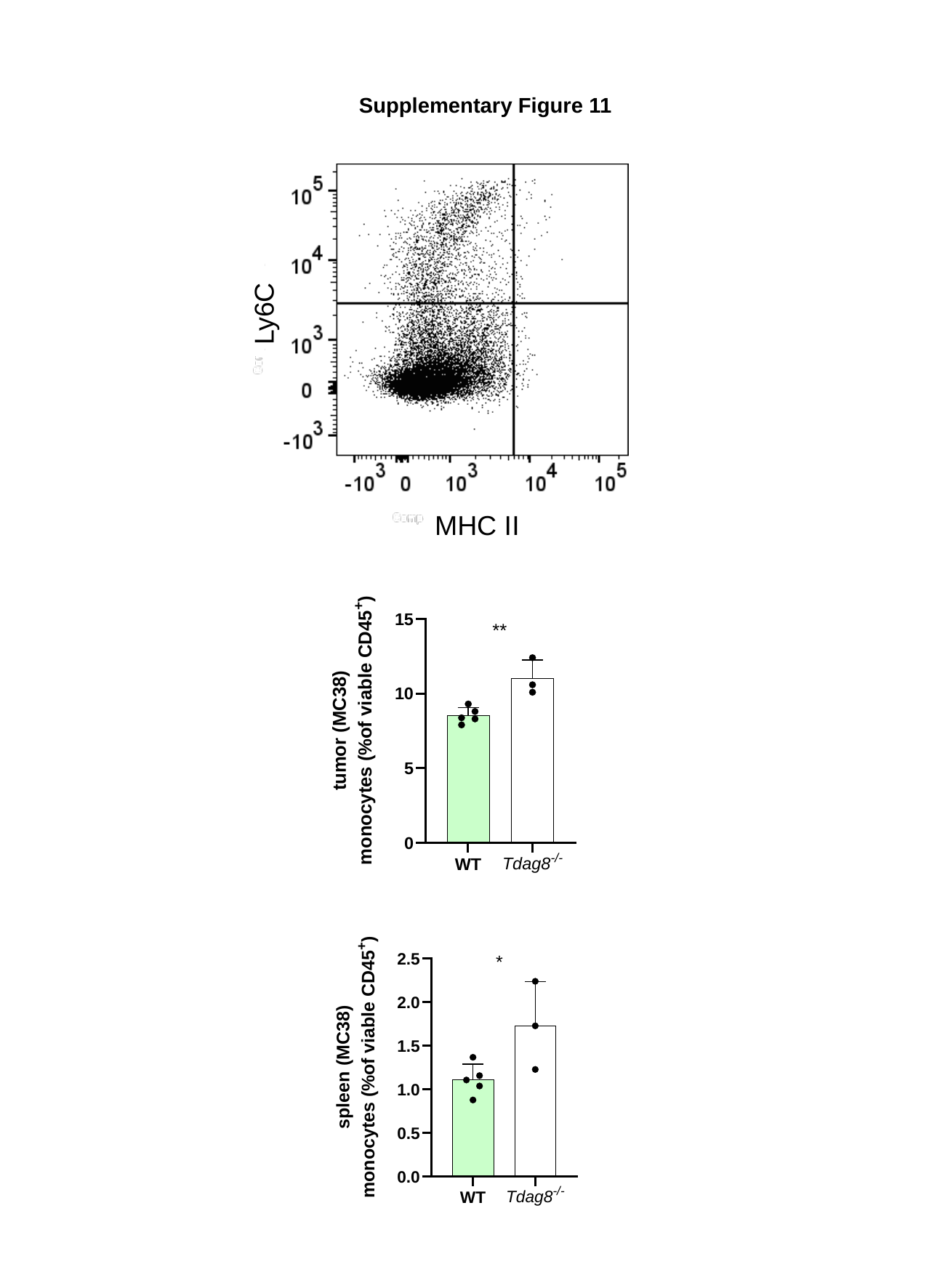

Supplementary Figure 11
Ly6C
MHC II
