# Supplementary material for: Loss of proton‐sensing TDAG8 increases tumor progression in mouse models of colon cancer
Source: Mol Oncol. 2026 Jun 9:10.1002/1878-0261.70283. Online ahead of print. doi: 10.1002/1878-0261.70283 (PMC13398714; doi:10.1002/1878-0261.70283)
Supplement: Supplementary file 2 — Table S1. Antibody panels used for flow cytometry. [file MOL2-9999-0-s002.docx]

**SUPPLEMENTARY TABLE**

| Panel | Fluorochrome channel | Target | Manufacturer | Reference number | Dilution | Host |
| --- | --- | --- | --- | --- | --- | --- |
| 1 | APC-Cy7 | viability marker | BioLegend | 423106 | 1:200 | NA |
| 1 | BV510, AmCyan | CD45 | BioLegend | 103138 | 1:400 | R |
| 1 | PE-Cy5 | B220 | Invitrogen | 15-0452-83 | 1:200 | R |
| 1 | BV785 | CD3 | BioLegend | 100232 | 1:200 | R |
| 1 | BV605 | CD11b | BioLegend | 101257 | 1:200 | R |
| 1 | AF647 | Ly6G | BioLegend | 127609 | 1:200 | R |
| 1 | BV711 | Ly6C | BioLegend | 128037 | 1:200 | R |
| 1 | PE-Cy5.5 | MHC II | BioLegend | 107626 | 1:200 | R |
| 2 | APC-Cy7 | viability marker | BioLegend | 423106 | 1:200 | NA |
| 2 | BV510, AmCyan | CD45 | BioLegend | 103138 | 1:400 | R |
| 2 | PE-Cy5 | B220 | Invitrogen | 15-0452-83 | 1:200 | R |
| 2 | BV785 | CD3 | BioLegend | 100232 | 1:200 | R |
| 2 | BV650 | CD4 | BioLegend | 100546 | 1:200 | R |
| 2 | PE-Texas red | CD8 | Becton Dickinson | 562283 | 1:200 | R |

**Table 1:** Antibodies used for FACS (M = mouse; R = rat; H = hamster; NA = not applicable).
